# Understanding how socioeconomic inequalities drive inequalities in COVID-19 infections

**DOI:** 10.1038/s41598-022-11706-7

**Published:** 2022-05-18

**Authors:** Rachid Laajaj, Duncan Webb, Danilo Aristizabal, Eduardo Behrentz, Raquel Bernal, Giancarlo Buitrago, Zulma Cucunubá, Fernando de la Hoz, Alejandro Gaviria, Luis Jorge Hernández, Camilo De Los Rios, Andrea Ramírez Varela, Silvia Restrepo, Norbert Schady, Martha Vives

**Affiliations:** 1 Universidad de Los Andes, Bogotá, Colombia; 2 Paris School of Economics, Paris, France; 3 Universidad Nacional de Colombia, Bogotá, Colombia; 4 Hospital Universitario Nacional de Colombia, Bogotá, Colombia; 5 Imperial College London, London, UK; 6 Pontificia Universidad Javeriana, Bogotá, Colombia; 7 Inter-American Development Bank, Washington, D.C USA; 8 World Bank, Washington, D.C USA

**Keywords:** Viral infection, Risk factors

## Abstract

Across the world, the COVID-19 pandemic has disproportionately affected economically disadvantaged groups. This differential impact has numerous possible explanations, each with significantly different policy implications. We examine, for the first time in a low- or middle-income country, which mechanisms best explain the disproportionate impact of the virus on the poor. Combining an epidemiological model with rich data from Bogotá, Colombia, we show that total infections and inequalities in infections are largely driven by inequalities in the ability to work remotely and in within-home secondary attack rates. Inequalities in isolation behavior are less important but non-negligible, while access to testing and contract-tracing plays practically no role because it is too slow to contain the virus. Interventions that mitigate transmission are often more effective when targeted on socioeconomically disadvantaged groups.

## Introduction

With around 464 million confirmed cases around the world as of March 2022, the COVID-19 pandemic has disproportionately affected disadvantaged groups. Evidence from multiple settings suggests that within each country, poor and minority groups are more likely to contract the disease^1–4^. In Bogotá, Colombia, we estimate in a companion paper that individuals in the lowest socioeconomic strata (SES) are 3.7 times more likely to have been infected with COVID-19 than those in the highest strata as of March 3rd 2021^5^. Addressing inequalities has been widely recommended to tackle the pandemic^6–8^. Some studies have documented factors that are likely to affect COVID-19 transmission patterns, including access to testing and contact tracing services^9–15^, biological factors related to susceptibility and infectiousness^16,17^, levels of exposure at work^18,19^, circumstances within the household^18–21^, lockdown and social distancing^10,14,22^, along with self-isolation behavior and compliance with regulations^14,23,24^. These factors are likely to differ by socioeconomic status, thereby driving inequality in COVID-19 infection rates across socioeconomic groups^15,18,25,26^. Optimal policy design will vary significantly depending on which of these factors is key: targeted policies that focus on high-risk groups will reduce both inequalities and overall transmission more effectively if they concentrate on the most important dimensions of inequality. However, few studies have so far been able to examine which factors are associated with socioeconomic inequality in COVID-19 infections and compare how important each one is for explaining overall inequality. We thereby provide the first study of a low- or middle-income country (LMIC) setting that (i) estimates multiple differences between socioeconomic groups in characteristics that are associated with inequality in COVID-19 infections, and then (ii) incorporates these differences into an epidemiological model to tease out their implied impact on the spread of the pandemic.

## Methods

### Differences in characteristics between socioeconomic strata

We use primary data from the CoVIDA project led by the University of Los Andes. This includes the results of 59,770 RT-PCR tests in Bogotá, targeted on a mostly asymptomatic adult population from the beginning of June 2020 to March 3rd, 2021. We combine this with administrative data from the Health Secretary of Bogotá (HSB) that covers all reported cases in Bogotá (Supplementary Materials S1.1 includes a detailed data description). Both datasets include information on individuals’ *socioeconomic stratum*, a classification commonly used as a proxy for economic welfare in Colombia. We use this six-level measure to create four SES groups for analysis, ranging from poorest to richest: 1&2, 3, 4, and 5&6.

Together, these data allow us to estimate a set of characteristics that are associated with infection rates, and to do so separately for each of the four socioeconomic groups. Table 1 displays the values of these characteristics for each group (and Table S7 includes standard errors, 95% CIs, source and estimation method). Throughout the paper, we classify the characteristics into 4 dimensions: (i) contacts outside of the household, (ii) contacts within the household, (iii) isolation behavior, and (iv) testing and tracing.

**Table 1:** Potential determinants of infection estimated by SES.

(a) All measures							
	Measure	SES Group				Full population	p-val, diff. between SES
Channel		1&2	3	4	5&6		
Infections outside home	Days working outside home (in last 14 days)	6.4	4.8	3.2	2.5	4.6	<0.001
	Number of non-work contacts outside home (in last 14 days)	1.108	1.392	1.506	1.423	1.314	0.063
	Secondary attack rate (outside home)	15%	13%	8%	12%	13%	0.2
	Contact matrix structure	[see Panel (b)]					
Infections inside home	Household size	2.99	2.81	2.50	2.48	2.84	<0.001
	Secondary attack rate (inside home)	26%	27%	24%	11%	26%	0.02
Isolation behaviour	Isolation rate after positive test result	0.87	0.85	0.86	0.87	0.86	0.61
	# days worked when has symptoms	3.03	2.29	2.4	1.5	2.6	<0.001
	# days worked when knowing about positive contact	4.5	3.4	3.5	2.2	3.9	0.016
	# days worked when someone is tested positive in same household	2.8	2.4	2.4	1.9	2.5	0.0040
Testing & tracing	Share detected among positive	11.7%	15.2%	22.2%	21.3%	16.1%	<0.001
	Test consultation delay in days	5.56	5.59	5.41	5.26	5.55	<0.001
	Test results delay in days	3.94	3.57	3.28	3.05	3.72	<0.001
	Average number of contacts traced	1.73	1.74	1.75	1.75	1.74	
	Proportion of infections that are contact traced	81%	84%	88%	89%	83%	
	Population size in Bogota	4,063,470	2,857,861	757,923	365,459	8,044,713	
	Sample size in CoVIDA Survey Data	22,171	31,636	14,608	7,539	75,954	
	Sample size wih PCR test in CoVIDA Data	15,818	24,450	11,759	6,158	58,185	

(b) Contact matrix							
	**Contact stratum**						
**Index case stratum**	1&2	3	4	5&6	Total		
1&2	206	98	14	2	320		
3	126	418	69	17	630		
4	9	52	58	25	144		
5&6	5	8	18	16	47		
Total	346	576	159	60	1141		

**Panel (a)**: The table displays variables that capture various determinants of infection, sorted in four categories, followed by population and sample sizes. It provides the average value for each SES and for the population all-together. The last column presents the p-value of the F-test of difference between the 4 SES. A p-value below 0.05 means that one can reject at the 95% confidence level that the variable has population average that is equal for all SES (two-sided test). Standard deviations, Confidence Intervals, data sources and explanations of the calculation methods are presented in Table S7. **Panel (b)**: The contact matrix enumerates the number of cases for each possible of combination of stratum of the index case and its contacts. Positive cases in the CoVIDA study were traced, from this data, We use the self-declared stratum of the index cases and all their non-household contacts to count the number of contacts within each cell.

The number of non-work-related contacts outside the home does not differ significantly across SES ($p= 0.06$). Secondary attack rate (SAR) for contacts outside the home also exhibits no significant differences across SES ($p=0.20$), with the overall average estimated at 13%. This result is consistent with our finding that self-declared protection practices are not systematically better among higher SES; lower SES even appear to compensate for their inability to remain at home by wearing masks and using antibacterials more frequently (Table S1). By contrast, there is a large and significant difference in the number of days working outside of home during the 14 days prior to the survey, which varies from 2.5 days for SES 5&6 to 6.4 days for SES 1&2 ($p<0.001$). This substantial difference is likely to reflect the well-documented variation in the ability to work remotely^27^.

Characteristics related to infections inside of homes also reveal differences. First, mean household size shows modest variation, from approximately 2.5 in wealthier households to 3 in poorer households ($p<0.001$ for the difference). Lower SES individuals therefore have more contacts within the household, which is known to be a particularly important setting for transmission^20^. There is a substantial difference in the SAR within household ($p=0.02$), ranging from only 10% in SES 5&6 to around 27% in SES 1–3. Corroborating this result, the positive correlation between household size and infection probability is stronger for lower SES (Figure S9b), which may partly be explained by more crowded housing conditions, since the poor have fewer rooms per household (Figure S9c).

Self-reported isolation for individuals who have been tested positive is high (86%) and does not vary significantly by SES ($p=0.61$). Other high-risk circumstances that require isolation, such as experiencing symptoms, lead to a substantial reduction in days worked outside of home for all groups. But richer groups are able to restrict their working activity in these circumstances significantly more than poorer groups.

Finally, access to testing and tracing could also affect infections if it leads to effective quarantine and isolation. We find differences in testing and tracing characteristics across SES. These differences may plausibly be explained by variation in health service quality that is correlated with income^28^. The likelihood of being detected conditional on being infected varies substantially, from 11.7% in the SES 1&2 to over 20% in SES 4, 5 and 6. There are moderate differences in the average delay in test consultations and results, which sum to 8.3 days for SES 5&6 and 9.5 days for SES 1&2. But average delays across *all* groups are very long. They clearly exceed recommendations for an effective Test, Trace and Isolate strategy, which suggest delays of no more than 5 days from onset of symptoms to the results of the test^11^.

### A theoretical model that emphasizes differences between socioeconomic groups

In order to quantify how the differences shown in Table 1 translate into differences in COVID-19 infection patterns, we use the results as inputs for a novel branching-process model of the spread of COVID-19. (See Tables S4 and S5 for a complete list and description of the model parameters.).

The model is stochastic and individual-based, building on early modelling work of the pandemic^10,11,22^. We structure the model by SES, allowing all parameter values found in Table 1 that are significantly different at the 5% level to be SES-specific.

**Figure 1:**
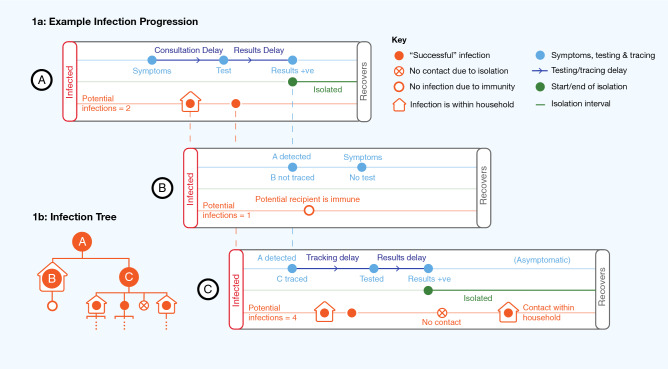
Visual representation of the theoretical model. An initial infection A potentially infects two other individuals, called B and C. (**a**) A successfully infects B and C. A gets tested upon experiencing symptoms, and isolates upon receiving a positive test result. This begins a process of contact tracing, through which C (but not B) is tested. B does not infect anyone else; the only person they potentially infect is immune. C infects two other people before being isolated. Once C is isolated, she does not come into contact with a potential infection outside the household, but still infects an individual in the same household. Individuals in the model may or may not be symptomatic, get tested, be contact traced, and they may isolate for a variety of reasons. (**b**) The infection tree summarises the “branching process” in the model, i.e. the first and second generation potential infections caused by A.

An example of a transmission process is shown in Fig. 1, and a second more detailed example is found in Fig. S10. The figures demonstrate some of the realistic features of the model, which include a distinction between contacts within and outside the home, assortative mixing, symptoms, testing, contact tracing, isolation, immunity, along with realistic distributions for all stochastically-generated timings.

The key implicit assumption underlying our analyses is that each of the parameters has a direct causal effect on COVID transmission. The size of this direct effect for each parameter, and the interactions between each parameter, are determined by the specific modelling assumptions about the transmission mechanism (for example, exactly how isolation reduces transmission, the length of the incubation period, and the household structure). These assumptions are discussed in detail in Sect. S1.2. This limitation should be taken into account, particularly when interpreting counterfactual results based on varying parameter inputs.

**Figure 2:**
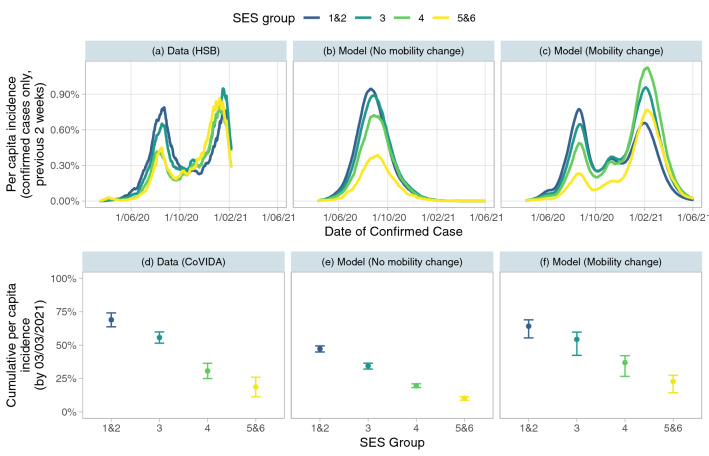
Estimations of incidence rate using data and baseline simulations. Panels (**a**), (**b**), and (**c**) show the per capita incidence over the previous 2 weeks based on *confirmed cases* (those who test positive) for each SES at each date. Panel (**a**) is based on the administrative data from the HSB on the number of confirmed cases at each date. Panel (**b**) is calculated using the number of infected individuals that test positive in the model simulation with no mobility change, while panel (**c**) uses the same calculation for the model simulation that allows for mobility to change over the course of the epidemic (in a way that best predicts total detected cases). Panels (**d**), (**e**) and (**f**) show the cumulative per capita incidence (including *both* confirmed and unconfirmed cases) by the 3rd March 2021 (the most recent date for which the CoVIDA data is available). Panel (**d**) uses positivity in CoVIDA data to calculate incidence, see Section S1.1. Panel (**e**) and (**f**) includes all infections in the versions of the model without and with mobility change respectively. All model results are calculated by taking the median value over 50 simulations. Model and actual dates are aligned by taking the model time period for which the model-predicted 2 week total per capita incidence is the same as the actual value on June 1st 2020, and setting this time period to be June 1st 2020.

Our baseline simulation of the epidemic uses the parameters as described in Tables S4 and S5. Figure 2 shows the infection patterns in each SES, both using data from Bogotá (panels (a) and (d)) and comparing to the results of our model in the baseline scenario. We use two variations of the model. In the first (panels (b) and (e)), the average number of out-of-home contacts for each group stays constant over the course of the epidemic, leading to a one-wave pattern. In the second (panels (c) and (f)), we account for changes in mobility over time by scaling the number of out-of-home contacts by a time-varying constant, calibrated to match total confirmed incidence (see Supplementary Materials Section S1.3.4). This constant is the same for all groups, implying that all predictions of inequality result from the differences in characteristics described in Section 2.1, rather than the calibration process.

Panel (a) displays the per capita incidence over the preceding 2 weeks for each group based on data on *confirmed* cases from the Health Secretary of Bogotá. We see evidence of inequality between groups, particularly in the first wave, where SES 1&2 reaches a peak incidence rate of 0.72%, around double the level of SES 5&6. The model predictions of confirmed cases, seen in panels (b) and (c), match this observed pattern relatively well, with more detection of cases in the lower SES. The observed pattern of detected cases lies within the range of the confidence intervals of both models for all groups in first wave (Fig. S11).

We also show that the model predictions match estimated inequalities in *true* infections, estimated with the CoVIDA data, which is a sample of mostly asymptomatic individuals and is thus less likely to be biased due to differential propensity to be tested. Panel (d) shows that the inequality in estimated true infections is even starker than that of confirmed cases: cumulative per capita incidence (during the entire period of the study) varies from 69% in strata 1&2 to only 19% in strata 5&6. Because the model with no mobility change (panel (e)) only captures the first wave of the epidemic, it underestimates the cumulative incidence rate in all groups, but it yields a prediction of the proportion of total cases that come from each SES (a proxy of inequality) that matches the CoVIDA data well (Fig. S12). When accounting for mobility changes (panel (f)), the model gives very similar predictions to the CoVIDA data estimations, despite mobility being calibrated on only aggregated confirmed cases, with a difference of 41 percentage points in cumulative per capita incidence between the lowest and highest SES. Broadly, we are able to predict the macro-level differences in infections well by introducing micro-level inequality into our epidemiological model.

### Ethics statement

All methods were carried out in accordance with relevant guidelines and regulations. All experimental protocols were approved by Universidad de Los Andes and the Secretaría de Salud de Bogotá. Ethics approval was obtained from the ethics committee of Universidad de los Andes (Act number 1278 of 2020). The ethics committee approved that the participants would receive the information via telephone and give their informed verbal consent, in order to comply with physical distancing and limit the restriction for a study is part of a public health surveillance strategy implemented jointly with the Health Secretary of Bogotá.

## Results

### Effects of reducing inequality on virus incidence

**Figure 3:**
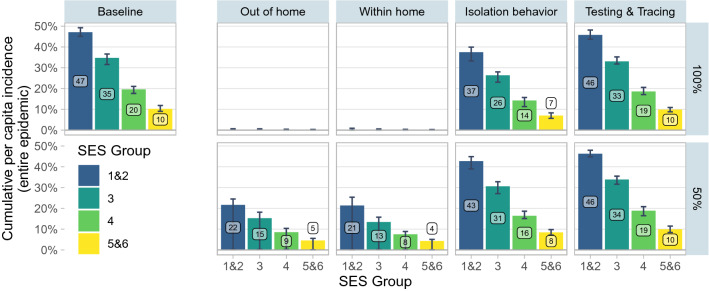
Upward Adjustment Scenarios. *Baseline* indicates the model with the parameters of Table 1 and no adjustment. The panels in columns 2 to 6 are the results of upward adjustment scenarios. In the top row of columns 2 to 6 (100% adjustment), the set of parameters indicated in the column heading is adjusted so that all SES have the same value as SES 5&6. In the bottom row (50% adjustment), all SES other than 5&6 have their parameters adjusted to move halfway to the value of 5&6. Parameters adjusted in each set are as follows: *out of home* (number of contacts outside the home), *within home* (within-household SAR, household size), *isolation behavior* (probability of isolating conditional on observing symptoms, testing positive, being contact traced, and probability of quarantining as a household), *testing & tracing* (probability of self testing, delay in test consultation, delay in test results, and probability of being contact traced). Point estimates denote the median of 50 simulations. Error bars indicate the 0.025 and 0.975 quantiles of the 50 simulations. When the error bars are close to 0, this indicates that some simulated epidemics die out in the very early stages, leading to incidence close to 0.

In order to identify the key channels that are associated with overall inequality in infections between SES, we examine the effects of simulating a reduction in inequality along 4 dimensions: (i) contacts outside of the household, (ii) contacts within the household, (iii) isolation behavior, and (iv) testing and tracing. For each of these dimensions, we first simulate a “100% upward adjustment” scenario, in which the characteristics of all SES are set at the level of the highest SES (5&6), and then a “50% upward adjustment” scenario (Fig. 3), in which the differences with respect to SES 5&6 are reduced by half.

First, we find that in a simulation in which all SES have as few contacts outside the home as SES 5&6, the epidemic collapses, with a median cumulative incidence of less than 1% across all SES (column 2). Under the assumptions of the model, if every SES had the ability to work on average only 2.5 days every 2 weeks outside of home, then the ${\mathcal {R}}_t$ would fall below one, leading to a containment of the virus from early stages. When we adjust by only 50%, there is still a marked reduction both in infections across all groups and in the inequality between groups. The lower bound on these simulations always includes a value less than 1%, since there are simulations in which the epidemic dies out in the early stages. This indicates that the differences in out-of-home contacts are strongly associated with overall inequality in COVID-19 infections between groups.

Second, attributing the within-home characteristics of strata 5&6 to all strata (column 3) leads to a reduction in infections that is as strong as the effect in the out-of-home scenario. Further analysis demonstrates that this effect is mostly driven by differences in the within-household SAR, while inequality in household size plays a significant but smaller role (Fig. S9). If the assumptions of the model are correct, there would be large potential benefits to policies that reduce within-home transmission for groups with crowded housing conditions, for example through recommending mask usage and social distancing within the home.

Third, we consider scenarios in which lower SES are just as able to isolate as SES 5&6 in high-risk circumstances (being in contact with or in the same household as a known case, or when presenting symptoms) (column 4). This leads to moderate simulated reductions in infections and inequalities: for example, cumulative incidence among SES 1&2 is reduced by 8 percentage points in the 100% scenario. Differences in isolation behavior are thus important, but are less associated with overall inequality in infections than the two previous channels. Reducing inequalities in isolation may nevertheless be more tractable for policy than changing characteristics like housing conditions or job-types. For example, using financial compensation to enable lower-income individuals to stay at home when symptomatic may be an effective strategy.

Finally, the simulated effect of improving access to testing and contact tracing among low SES to the level of SES 5&6 has an effect on infections that is not significantly different from 0 (column 5). This is true despite the substantial inequality in access (see in Table 1). The absence of effect can be explained by the fact that, on average in Bogotá, delays in accessing testing, receiving results, and being contact traced are so severe *across all groups* that testing and tracing has little effect at all in mitigating the spread of the virus in this context.

**Figure 4:**
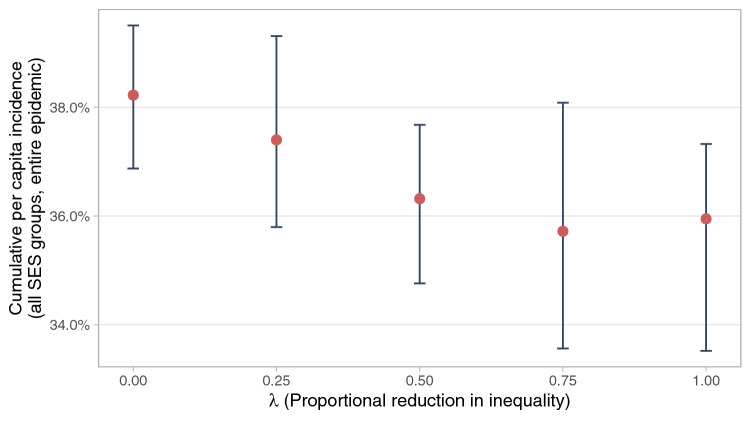
Mean-preserving reduction in inequalities. Describes the effect of reducing inequalities in all parameters simultaneously while preserving the mean of all parameters. The value of parameter *k* for an SES *j* in the baseline simulation can be written as $v_{jk} = {\overline{v}}_k + \varepsilon _{jk}$, where ${\overline{v}}_k$ is the (population weighted) mean value for the parameter across all groups, and $\varepsilon _{jk}$ is some deviation. The graph plots the results of adjusting all parameters to the value $v^*_{jk}(\lambda ) = {\overline{v}}_k + (1 - \lambda ) \varepsilon _{jk}$. The outcome variable is the median cumulative per capita incidence across all SES over the course of the entire simulated epidemic in 50 models with no mobility change. Error bars indicate the 0.025 and 0.975 quantiles of the 50 simulations.

To further examine the role of socioeconomic inequality, we simulate the effect of a pure reduction in inequalities. Specifically, we reduce the *dispersion* of all the characteristics that were found to be significantly different across SES while preserving the *mean* of each variable (Fig. 4). We find that if these inequalities are fully collapsed, total infections would be reduced from 38.2% to 35.9% of the population. The simulated effect is moderate but statistically significant ($p<0.001$). Inequality *in and of itself* is associated with more widespread infection, even when holding constant the average characteristics of the population.

### Alternative policy scenarios

**Figure 5:**
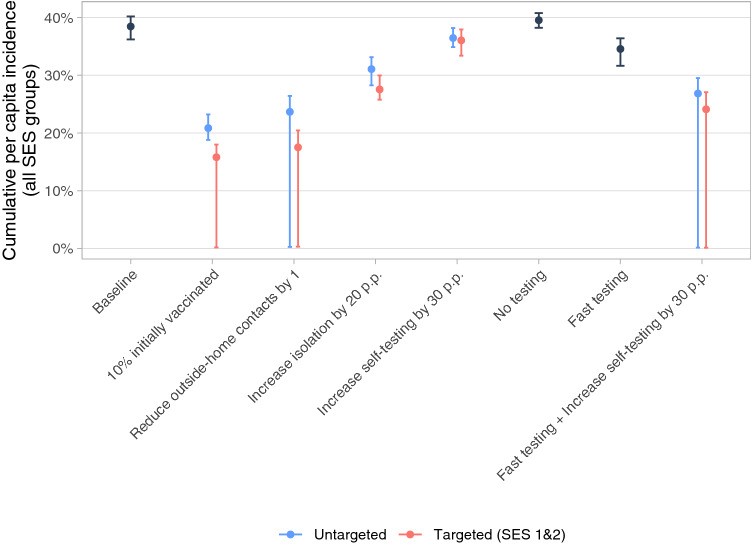
Policy-style scenarios. In “Untargeted” scenarios, policy adjustments affect all groups equally. In “Targeted” scenarios, only the parameters of SES 1&2 are adjusted, but adjustments in this group are greater, such that the mean adjustment across the whole population is the same as in the untargeted scenario. “10% initially vaccinated”: 10% of the population are immune to the virus from the start of the epidemic. “Reduce outside-home contacts by 1”: mean reduction of 1 in contacts outside the home. “Increase isolation by 20 p.p.”: mean increase of 20 percentage points in probability of isolating conditional on being symptomatic and being contact traced. “Increase self-testing by 30 p.p.”: mean increase of 30 percentage points in the probability of being tested after observing symptoms. “No testing”: probability of self-testing and of being contact traced are set to 0. “Fast testing”: all tests have a consultation delay and a results delay of 1 day. Outcome variable is the median cumulative per capita incidence across all SES for 50 simulated epidemics with no mobility change. Error bars indicate the 0.025 and 0.975 quantiles of the 50 simulations.

We next examine simulations of policy-style scenarios that indicate (i) what types of policies may be effective in combating the epidemic, and (ii) whether targeting policies on low socioeconomic groups can reduce the spread of the virus. Figure 5 shows the main results, while Fig. S17 shows the results when varying the intensity of each policy. The first set of simulations describes the results of (i) 10% population immunity (e.g., due to vaccinations at an early stage), (ii) a reduction of 1 in outside-home contacts (e.g., due to restricting economic activity, or a policy facilitating or enforcing remote work), and (iii) an increase in ability to isolate (e.g., due to financial support for those required to isolate). Increasing immunity and reducing out-of-home contacts lead to large reductions in simulated infections; increasing isolation leads to more modest effects. Even when holding constant the number of beneficiaries, reductions are between 28% and 49% larger when targeted on the lowest SES ($p<0.001$ for all differences). If the assumptions of the model hold, policies that target socioeconomically disadvantaged populations are likely to be substantially more effective.

By contrast, increased access to testing for symptomatic individuals has only a small impact on total infections, and this impact does not differ significantly if targeted or not. Access to tests is not associated with lower infection rates in Bogotá, likely because of severe delays across all SES, and because initial testing coverage is limited.

In keeping with this claim, even a complete removal of testing and tracing (the “No testing” scenario) only increases the overall simulated incidence rate by a mean of 1.0 percentage points ($p < 0.001$), while increasing the speed of testing to an average of 2 days from symptoms to detection results in a modest 4 percentage point reduction in incidence rate from the baseline scenario. Combining this “Fast testing” scenario with an increase in the probability of being tested leads to more substantial reductions in transmission, with the overall incidence rate reducing to below 30%. Targeting the improvements in self-testing on SES 1&2 leads to an additional mean reduction of 2.7 percentage points compared to the non-targeted scenario (*p*-value of the difference$=0.04$).

## Discussion

This paper documents differences across SES along multiple dimensions that are relevant to the spread of COVID-19. We provide one of the first studies that estimates the relative importance of each form of inequality for explaining disparities in COVID-19 infections. Such estimates are important to improve policy design. Disparities in types of jobs and the ability to work from home are shown to be a key factor associated with inequalities. Important channels that have seen less emphasis in existing research include within-home SAR and the ability to isolate when required (in particular when one has symptoms, a detected individual in the household, or a recent contact with a person tested positive). Finally, while poor individuals do have substantially less access to tests, and a lower chance of being detected and traced, this does is not associated with inequalities in incidence rates, probably because testing and tracing in Bogotá is too slow to contribute to the mitigation of the virus.

We find that simulating an improvement in the conditions of lower SES translates to incidence reductions that are approximately proportional across all groups. Our model allows for assortative mixing, in which each group is somewhat more likely to contact their own group. Despite this, even the highest SES benefit from improvements of the conditions faced by the lowest SES. Our results suggest that worse conditions for disadvantaged groups are associated with worse outcomes for all.

Even when maintaining the same mean characteristics across the whole population, a reduction in inequality reduces the simulated spread of the virus. In addition, simulated policies are more effective at reducing transmission when they target disadvantaged socioeconomic groups. There may be similar benefits of targeting for real-world policies. More generally, some factors in the model are more strongly associated with transmission reductions. Our results suggest placing a particular emphasis on (i) maximising the ability to work remotely for lower socioeconomic groups where possible, or temporary and targeted economic shutdown measures in the absence of other alternatives, and (ii) raising awareness that within-home infections are a major source of transmission, but that this transmission may be avoidable, and may be mitigated using within-house preventative measures such as mask use. Immediate financial compensation for individuals required to isolate, including close contacts, housemates of infected persons, and anyone with COVID-19-related symptoms, may also be a tractable policy lever. Our model shows a negligible relationship between inequality in infections and test-and-trace access. This suggests that although testing systems provide valuable information about the spread of the virus, they may be so slow that they have little mitigating effect on the transmission. In such cases, policymakers must be transparent about testing delays, and consider the possible benefits of a dramatic speed-up of the testing system if this is feasible.

There are a number of important limitations of this study. First, there may be other unobserved variables that are correlated with both the model inputs and the outcome variable (virus incidence). Our model makes assumptions to infer how virus transmission would change if the model inputs were to change. But if these assumptions are wrong, or fail to take into account other correlated factors, then these inferred causal relationships will be biased.

For example, our model does not incorporate differences in the age distribution between SES. As shown in Table S6, the lower SES have a younger population and, on average, younger individuals tend to have higher COVID-19 positivity rates. Since we do not explicitly incorporate these age differences between SES in our model, we may be overestimating the role of the model factors in explaining inequality in infections between groups. There may be a number of other unobserved factors that cause a similar bias, such as individual preventative measures (e.g., mask use, handwashing), use of public transport, transmission network structure, and the role of schools and children. Nevertheless, Fig. S12 indicates that the model predicts overall inequality well.

Second, the correlations between unobserved and observed variables may vary over time. This implies that any bias in our results may not be constant over the study period. It also limits our ability to generalise our results to the phase of the pandemic after our study period, particularly as we focus on the early period of the pandemic when lockdown restrictions were particularly severe. For example, our model does not account for differential effects across SES of subsequent mobility changes.

Third, the counterfactual scenarios we examine do not account for endogenous mobility reactions, leading to overly extreme results in which the virus is completely contained. Since the model starts in the conditions of generalised lockdown in Bogotá, with $$R_q = 1.22 $$, any scenario sufficient to reduce this number to below 1 will lead to a total containment of the epidemic. In reality, mobility restrictions may have been loosened sooner if this was the case, leading to more infections than predicted by the model.

Fourth, the CoVIDA data we use is not fully representative of the Bogotá population, although it is one of the most comprehensive datasets available in Latin America, including both PCR and survey results.

Fifth, we do not examine infection mortality, which may differ by SES group. Inequality in mortality may be more extreme than inequality in infections (e.g., because of better quality hospital treatment in higher SES) or less extreme (e.g., because higher SES are older on average). Developing a model that combines differences between SES with age variation and mortality is a promising avenue for future research.

Sixth, our baseline model only accounts for a one wave epidemic. This permits a clear-cut examination of the drivers of inequality, but does not account for immunity effects that become important in later waves. When using a model that allows for mobility change, the upward adjustment scenarios generate two epidemic waves (Fig. S16), in which any reductions in infections in the first wave are offset by lower immunity levels that lead to much larger second waves. Such a model is not necessarily more realistic, since it assumes that mobility would not have been restricted even in cases of extremely high incidence rates in the second wave. Future research should therefore prioritise models that incorporate both inequality and endogenous mobility reactions. Nevertheless, the results imply that in the absence of widespread vaccination, measures that reduce infections may only delay an epidemic rather than prevent it.

Our findings provide new evidence on the importance of different channels that are associated with inequalities in COVID-19 infections. Our model simulations suggest that improving the circumstances of the most disadvantaged groups, including by targeting interventions on the poor, may have benefits for all. Socioeconomic inequalities should be taken into account in order to better handle both the COVID-19 crisis and potential future epidemics.

## Supplementary Information

Supplementary Information.

## Data Availability

All the code and data used for this study is available at the following GitHub repository: https://github.com/dmbwebb/covid_model.
