# Supplementary material for: Understanding how socioeconomic inequalities drive inequalities in COVID-19 infections
Source: Sci Rep. 2022 May 18;12:8269. doi: 10.1038/s41598-022-11706-7 (PMC9117199; doi:10.1038/s41598-022-11706-7)
Supplement: Supplementary file 1 — Supplementary Information. [file 41598_2022_11706_MOESM1_ESM.pdf]

SUPPLEMENTARY MATERIALS for Understanding how socioeconomic inequalities drive inequalities in COVID-19 infections

Rachid Laajaj[*†1], Duncan Webb[†2], Danilo Aristizabal[1], Eduardo Behrentz[1], Raquel Bernal[1], Giancarlo Buitrago[3, 4], Zulma Cucunubá[5,6], Fernando de la Hoz[3], Alejandro Gaviria[1], Luis Jorge Hernández[1], Camilo De Los Rios[7], Andrea Ramírez Varela[1], Silvia Restrepo[1], Norbert Schady[8], and Martha Vives[‡1]

[1]Universidad de Los Andes, Bogotá, Colombia
[2]Paris School of Economics, Paris, France
[3]Universidad Nacional de Colombia, Bogotá, Colombia
[4]Hospital Universitario Nacional de Colombia, Bogotá, Colombia
[5]Imperial College London, London, UK
[6]Pontificia Universidad Javeriana, Bogotá, Colombia
[7]Inter-American Development Bank, Washington D.C, United States
[8]World Bank, Washington D.C, United States

[*]Correspondence and requests for materials should be addressed to Rachid Laajaj (r.laajaj@uniandes.edu.co)
[†]These authors contributed equally to this work.
[‡]On behalf of the CoVIDA working group.

S1 Materials and Methods

S1.1 Data Description

S1.1.1 CoVIDA Data

Our primary data comes from the CoVIDA project led by the University of Los Andes. This community-based sentinel surveillance initiative was integrated with the district's public health surveillance and organized by occupation group. The CoVIDA project was designed to help contain the spread of COVID-19 through active surveillance among mostly asymptomatic individuals and to provide a range of information that differs from the self-selected symptomatic individuals tested in health facilities. The sample includes 59,770 RT-PCR tests of COVID-19 on 55,078 different individuals in Bogotá from the beginning of June 2020 to March 3rd, 2021. At the time of registration, individuals were surveyed to capture various characteristics, including occupation, socioeconomic stratum, and address.

Two main strategies were employed to recruit participants, and approximately one half of the total sample comes from each strategy. First, through 74 agreements with institutions and companies, we obtained long-lists that we used to contact and invite participants. Most lists were specific to a given occupation, based on the employees of a large company or on a list of individuals who were signed up to a specific mobile app. We also used some lists of residents based on beneficiaries of social programs. We randomly selected participants from the lists and contacted them to invite them to be tested for free. The total population of all lists covers 20% of the population in Bogotá. This means that it is relatively close to a population-based sampling, but with an over-representation of some occupations that were prioritized in the CoVIDA project, in particular because they were expected to be more exposed (which is why we re-weight by occupation to maintain representativity of actual occupations in Bogotá).

The second source of participants' identification comes from public announcements made by the CoVIDA team through various communication channels to invite people to be tested, stating explicitly that the invitation is open to those that are asymptomatic.

For estimations of cumulative incidence with the CoVIDA data, we convert the positivity rate of CoVIDA tests into a number of daily new cases. To do this, we take into account the estimated sensitivity of the RT-PCR tests, which implies that individuals can be tested positive for a period of 17 days on average [1]. Hence in order to obtain the number of cases per day and per inhabitant, one needs to divide the positivity rate by 17. Intuitively, if, any person that gets infected can be tested positive during 17 days on average, then the positivity rate should be 17 times higher than the numbers of daily new infections. See our companion paper [2] for more details on this calculation.

Our second database comes from administrative records, collected by the Health Secretary of Bogotá (HSB, in Spanish the *Secretaria de Salud de Bogotá*), that cover the universe of cases of Bogotá residents that have been tested positive to COVID-19 by any laboratory using an RT-PCR test, starting from the beginning of the pandemic (January 23rd, 2020) until February 14th, 2021. All laboratories in Bogotá must report any positive test to the HSB, which in turn reports

it to the National Health Institute that provides national statistics used by the World Health Organization. This administrative data also comes with basic socioeconomic characteristics from a form that is a mandatory part of the institution's report when recording a positive case to the HSB.

Both databases include information on an individual's *socioeconomic stratum*, a classification that is based on the neighbourhood of residence and is commonly used in Colombia as a proxy for the household's economic living conditions. Neighborhoods are categorized into one of 6 levels, where 1 is the poorest and 6 is the wealthiest. To gain power, we pool together strata 1 and 2 and strata 5 and 6, leaving us with the four following groups: SES 1&2, 3, 4 and 5&6.

S1.2 Model details

In the model, each infected individual comes into contact with other individuals and potentially infects them. An example of this process is seen in Figure 1. A potential secondary infection is defined as a contact that would become infected unless prevented by immunity or isolation. The stochastic number of potential secondary infections each infected person generates depends on (i) whether they are symptomatic or asymptomatic, (ii) the number of contacts they have during their infectious period, and (iii) the secondary attack rate. We distinguish between contacts within the household and outside the household (Figure 1 panel (b)) and each type of contact has a different secondary attack rate. All members within the household are assumed to be contacts and to be from the same SES. Contacts from outside the household are sampled randomly from the entire population with sampling weights that reflect our estimated contact matrix between SES (see Table 1b, Panel b). The model therefore permits assortative mixing; contacts are typically more likely to occur within a SES, but are not restricted to the same SES.

Potential infections may not translate into actual infections for two reasons. First, because of immunity, a potential secondary case that has already been infected in the past will not develop into a new infection (e.g. person C). Second, any out-of-home contact is prevented if the individual is isolating at the time of the potential infection (while within-home transmissions are unaffected by isolation). An individual may isolate for four reasons: (i) after experiencing symptoms, (ii) after receiving a positive test result, (iii) after being contact-traced, or (iv) if the entire household quarantines because one member received a positive test result.

An individual may be tested either because they decide to take a test themselves after experiencing symptoms (person A and person E in Figure 1), or because they are contact traced. An individual who is "detected" (tests positive) is subjected to a process of contact-tracing with a probability lower than 1, leading to the possible testing and detection of each of their secondary cases. For example, in Figure 1, A is detected, leading to B and D (but not E) being traced and tested.

The model is based on a branching process structure. We initiate the model with 1/5000 of the population infected, and iterate over one-period cycles (that are equivalent to one day). In each period, infected individuals can transmit the virus to others, and other events such as isolation, testing, and tracing can also occur. These events, and how they are simulated, are de-

scribed in detail below. In all results, the model is simulated with a total population of 100,000, and the proportion of initial cases in each SES is set to be equal to the SES's population as a proportion of total population. The parameters used in the model are summarised in Table S4, which describes the parameters common across all SES, and in Table S5, which describes the parameters that were potentially allowed to differ by SES.

S1.2.1 Secondary Infections

Infected individuals come into contact with other simulated individuals. Contacts are divided into two types, "household" and "external" (outside the household). The number of contacts within the household for individual i, δ_i^{hh}, is assumed to be equal to $(householdsize_i - 1)$. The number of contacts outside the household for an individual i, δ_i^{ext}, is drawn from a negative binomial distribution with a dispersion parameter $k = 0.58$ (taken from [3]) and a mean of μ_j, where j is i's SES (see Figure S6). We then define the number of "potential" infections generated by i. This is the number of infections that would be generated by i, absent considerations of any isolation behavior or immunity. Some potential infections will not convert into actual transmission because the infector is isolating or because the potential infectee is immune. The number of potential infections that i generates in each category is given by a binomial distribution with size equal to the number of contacts and probability of infection from the group-specific secondary attack rate (SAR):

$$p_{ij}{}^{hh} \sim Binom(\delta_i^{hh},\ SAR_j^{hh})$$
$$p_{ij}{}^{ext} \sim Binom(\delta_i^{ext},\ SAR_j^{ext})$$

People in the same household are assumed to be from the same SES. External contacts are selected randomly from the wider population with sampling weights that depend on the estimated contact matrix seen in Table S5, panel (b). This allows for phenomena such as assortative mixing (i.e. individuals are more likely to encounter someone from their own group).

S1.2.2 Symptoms

Based on data from a recent review [4], we assume that 20% of infected individuals remain completely asymptomatic throughout the course of infection. We also follow this review in assuming that these individuals are less infectious than symptomatics, with a relative risk of 0.35 (i.e. the secondary attack rate is $0.35 \times SAR_j$). The remaining 80% of infected individuals present symptoms according to the timing described in the next subsection. Symptomatic individuals may get tested upon observing their symptoms (see the Section S1.2.5), whereas asymptomatic individuals will never get tested through this channel.

S1.2.3 Timings

The incubation periods of both infector and infectee are both assumed to be distributed lognormally with parameters drawn from a meta-analysis of the literature on the incubation period for

COVID-19 (μ = 1.63 and σ = 0.5) [5].

The serial interval is assumed to be gamma-distributed with parameters α = 8.12, β = 0.64 (and with the distribution function translated by Δx = -7.5 to allow for negative values), all taken from [6]. This distribution implies that 10.1% of serial intervals will be negative. The mean serial interval is 5.2 days.

If the incubation periods and the serial interval are assumed to be independent, then the implied generation interval is often significantly below 0, which is epidemiologically impossible. Therefore, to make the distributions of the incubation periods and serial interval consistent with a realistic distribution of the generation interval, we allow for correlation between the values of the two incubation periods and the serial interval. To do this, we first draw values from a trivariate skewed-normal distribution [7, 8], and then transform the resulting variables to follow the lognormal and shifted-gamma distribution described above. The variance-covariance matrix and the skew parameters of the trivariate skewed-normal are chosen to match the resulting generation interval distribution to a gamma distribution with a mean of 5.2 days (the same as the mean serial interval) and with a freely varying shape parameter. This ensures that the generation interval is realistic and that negative generation intervals are rarely drawn. (In approximately 0.36% of cases, generation intervals less than 1 day are drawn using this procedure. Secondary infections are assumed to only be possible 12 hours after infection, so in such cases we redraw the incubation period and serial interval values using the same procedure until the generation interval is greater than or equal to 1 day.)

The process that matches the the generation interval distribution to a gamma distribution is carried out by numerically minimizing the Kolmogorov-Smirnoff test statistic. The shape parameter of the gamma distribution is estimated to be 4.79. The mean of the generation interval distribution, 5.2 days, is consistent with a recent meta-analysis [9], which estimated the mean generation interval to be 4.8 [95% CI 4.3-5.41] when using a fitted gamma distribution. The estimation process results in a shape parameter of 4.79. The probability distributions of all timing variables can be found in Figures S2, S3, S4, and S5. All timing variables are assumed follow the same distribution for both household infections and external infections.

S1.2.4 Immunity and isolation

A "potential" infection in the model may not become an actual new infection for three reasons:

1. The potential infectee has already been infected. All individuals who have been infected once are assumed to be immune indefinitely.

2. The potential infector is isolating at the time of potential infection.

3. The potential infectee is isolating at the time of potential infection.

If the potential infectee or infector is isolating at the time of potential infection, then this reduces the probability of infection to 0 for out-of-home infections, while leaving the probability of within-home infections unchanged.

Potential infectors may isolate for one (or more) of 4 reasons:

1. Symptoms. Upon symptom onset, individuals will isolate with some probability that depends on their SES.

2. Positive test result. Upon receiving a positive test result (see below), individuals will isolate with some probability conditional on their SES.

3. Contact tracing call. Upon being told by a contact tracing team that they have been in contact with an infected individual, individuals will isolate with some probability conditional on their SES.

4. Household quarantine. A proportion ω_j of households in each group j are quarantining-types. This means that if at least one person in the household isolates because they receive a positive test result, then all the members of the household also isolate for the same period as the detected individual.

All the relevant probabilities can be found in Table S5.

Potential infectees also reduce transmission by isolating through the household quarantining channel, although this plays a minor empirical role in reducing overall transmissions in the model simulations. In most cases, isolating individuals stop isolating when they recover, which is assumed to be 10 days after experiencing symptoms. However, there are some cases in which individuals will "deisolate" before recovery. Deisolation occurs only in the case when these conditions hold: (i) individual was isolating due to a contact tracing call, (ii) the individual then receives a negative test result (either because they were tested before they were infected, or because the test was a false negative), and (iii) the individual is not isolating for any other reason. This feature of the model avoids overestimating the efficacy of contact tracing in cases where tests are carried out very quickly.

S1.2.5 Testing and Contact Tracing

There are two reasons for which an individual can be tested in the model:

1. "Self" testing. Upon symptom onset (if symptomatic), individuals will be tested with some probability conditional on their SES.

2. Contact tracing. If an infected individual from group j is "detected" (is tested positive), then everyone they infected has some probability π_{j} to be called by a contact tracing team and then tested themselves.

The relevant probabilities can be found in Table S5.

Tests are imperfectly sensitive, and test sensitivity is assumed to depend on time since infection, following the data from [10] (see Figure S1, in which test sensitivity increases rapidly over the first few days of infection and then decreases after around day 5.

Figure S1. Test Sensitivity (Source: Grassly et al 2020)

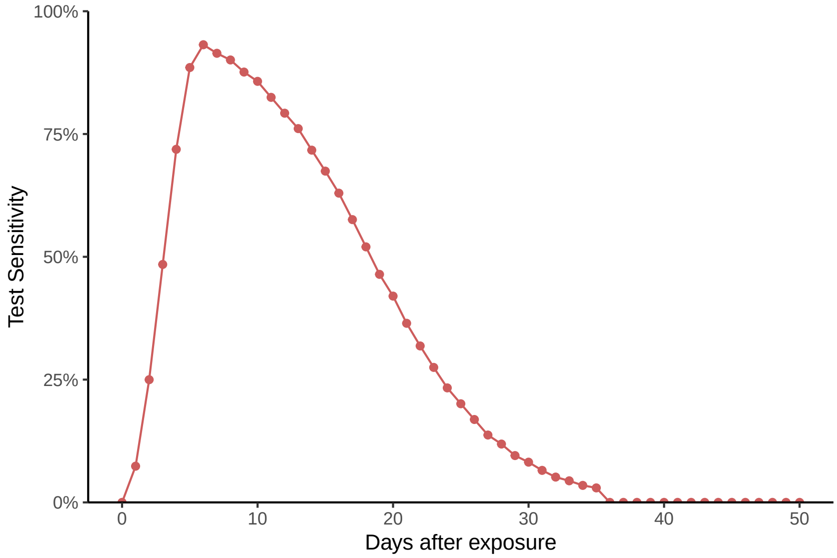

All testing processes include relevant delay times. The "test consultation delay" denotes the time it takes for individuals to access a test after symptom onset (if they self-test). The "test results delay" denotes the time it takes for results to be given to an individual after they are tested (for all three types of test). And the "contact tracing delay" denotes the time it takes to contact and test an individual after the original infector is detected (these are assumed to take place simultaneously).

S1.3 Calibration exercises for out-of-home contacts

In order to calculate the full matrix of out-of-home contacts to be input into the model, we first estimate the average number of non-work contacts outside the home for each SES j (from individuals traced in the CoVIDA project), and add it to contacts due to working outside of home, estimated from average number of days of work in a 14-day period for group (from the full data) j. Together, it provides the average number of contacts of an individual from group j, called μ_j. We then use the COVIDA contact tracing data to infer the proportion of the contacts of an individual from group j that come from group k, called q_{jk}. This basically assumes that people are "missing at random" i.e. probability of being missing is independent of the group. Together these pieces of information allow us to calculate the full symmetric contact matrix $\mathbf{\Delta}$ that describes the number of contacts in an average infectious period from group j to group k (absent any isolation behavior). The steps carried out are described in more detail below.

Figure S2. Incubation Period

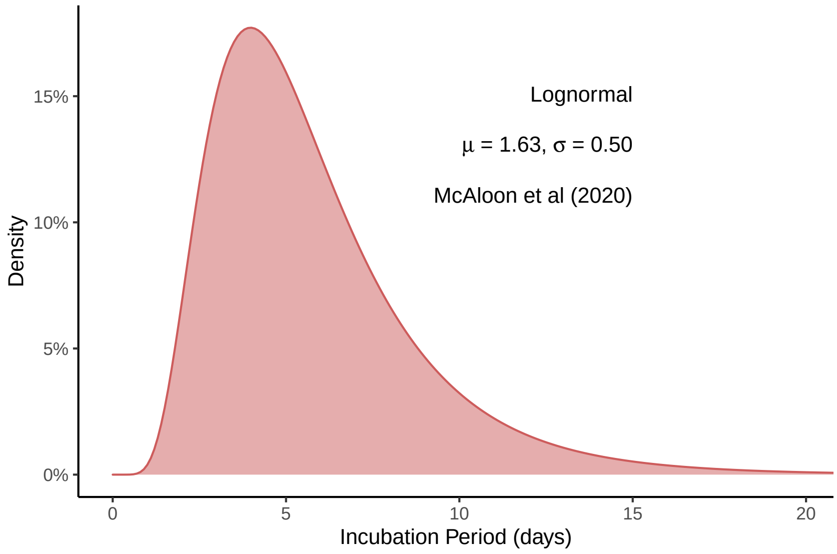

Figure S3. Serial Interval

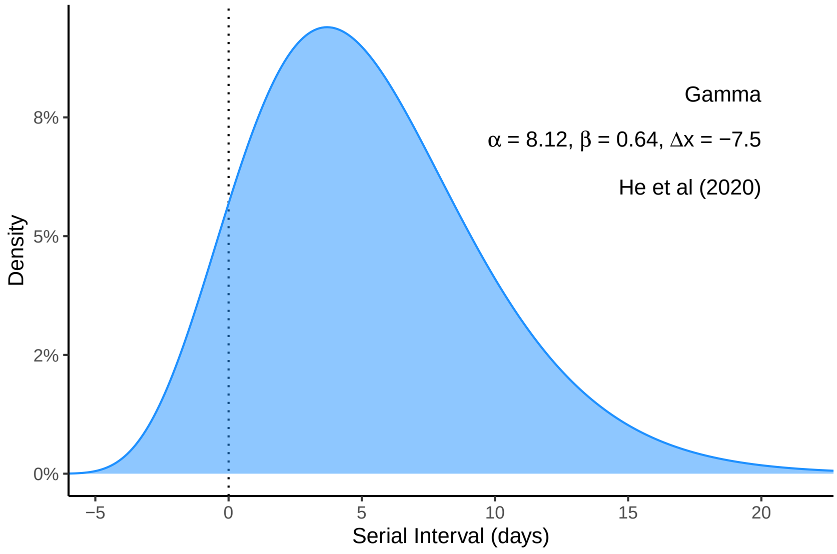

Figure S4. Implied Generation Interval

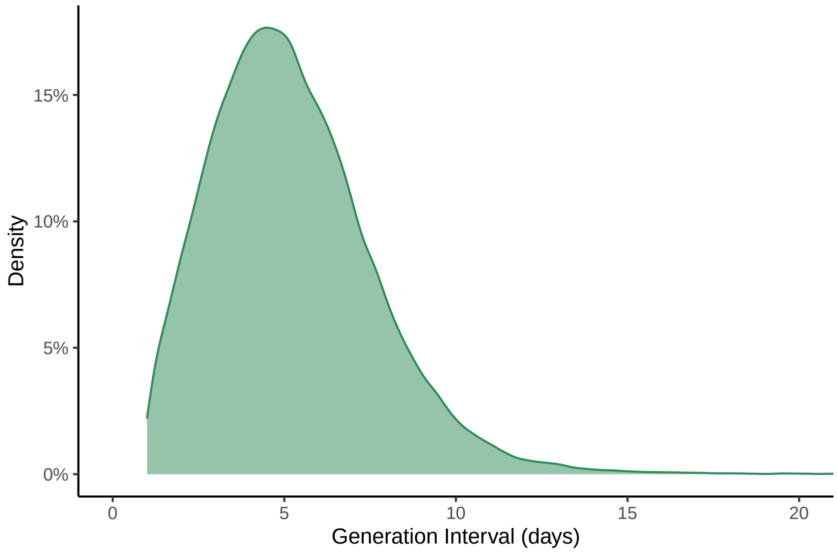

Figure S5. Implied Infectiousness Profile

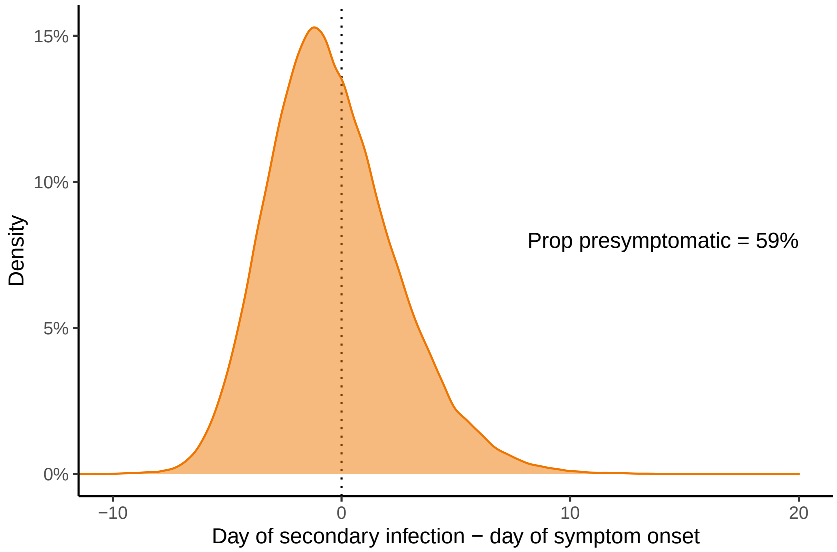

Figure S6. Contacts outside of home (distribution)

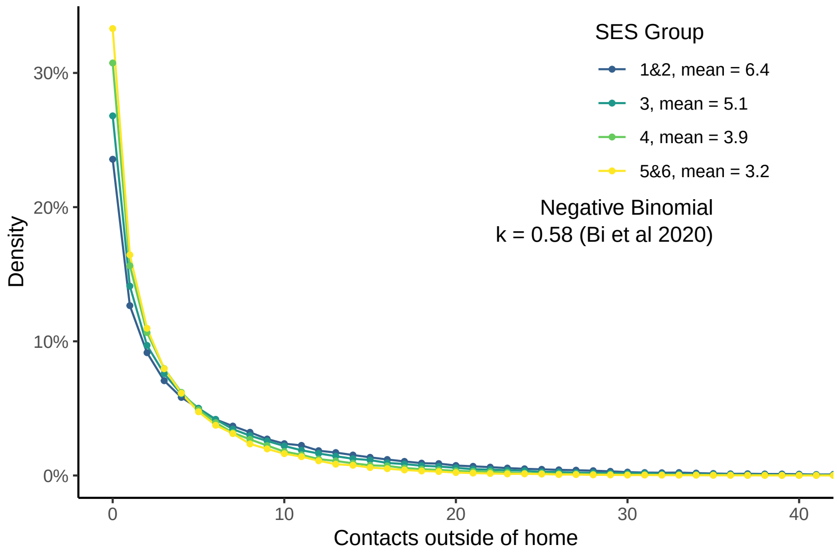

S1.3.1 Contacts outside the home

We assume that the mean number of contacts an individual from group j has outside of home during an average infectious period is given by μ_j, defined as:

$$\mu_j(\lambda) = v_j + \lambda w_j$$

Where v_j is the mean number of *non-work* contacts from outside of home for group j, and w_j is the mean number of days of work for group j. Both of these are estimated directly from the CoVIDA data (see Table 1) in the main text. λ is an unknown parameter (the "work factor") that describes the relationship between the number of days at spent at work and the number of out-of-home contacts during an infectious period (we assume this relationship to be linear). We choose a value of λ by calibrating the model to the true value of $\mathcal{R}_q$ estimated from the data. More specifically, we carry out the following steps:

1. For each candidate value λ_0 in the set of candidate values $\{0.05, 0.1, 0.15, 0.2, \ldots, 2.0\}$:
   (a) Calculate the implied value of $\mu_j(\lambda_0)$.
   (b) Use the values of $\mu_j(\lambda_0)$ to calculate the implied full contact matrix $\mathbf{\Delta}$ using the maximum likelihood process outlined in Section S1.3.2.
   (c) Run 50 simulations of the first 100 periods of the model.
   (d) Use these simulations to calculate the implied value of $\mathcal{R}_q$ for this value of λ_0 following the steps in Section S1.3.3. Call this $\mathcal{R}_q(\lambda_0)$.

2. Set λ equal to λ^* , where λ^* is the value of λ_0 that minimizes $|\mathcal{R}_q(\lambda_0) - \mathcal{R}_q^*|$, with $\mathcal{R}_q^*$ being the true value of the basic reproduction number calculated based on the real data using the methodology in Section S1.3.3.

3. Use $\hat{\mathbf{\Delta}}_{ML}(\lambda^*)$ as calculated using the method in Section S1.3.2 as the value for the contact matrix in the model simulations.

When we carry out the above steps in this way, we find that $\lambda^* = 0.8$, implying that the value for the work factor that best matches the observed growth in early cases in Bogotá's epidemic is 0.8. In other words, the model assumes that the mean number of out-of-home contacts during the average infectious period increases by 0.8 with each additional day of work outside of home.

Using this work factor, the estimated contact matrix based on the maximum likelihood procedure in Section S1.3.2 for a population of 100,000 is:

$$\hat{\mathbf{\Delta}}_{ML}(\lambda^*) = \begin{bmatrix} 272,155 & 49,440 & 5,162 & 2,432 \\ 49,440 & 102,967 & 16,864 & 2,729 \\ 5,162 & 16,864 & 8,431 & 6,022 \\ 2,432 & 2,729 & 6,022 & 5,247 \end{bmatrix}$$

The estimated $\mathbf{\Delta}$ matrix tells us the number of contacts that occur between each pair of groups in an average infectious period (which is the same across all groups). For example, the value in the first column and second row indicates that 49,440 contacts occur between individuals of groups 1 and 2 (corresponding to SES 1&2 and 3 respectively) during an average infectious period. Contacts are assumed to be mutual, so that the matrix is symmetric. We can see from the estimation result that there is strong assortative mixing: each group is more likely than random to contact another individual from the same group. (Note that smaller values for the 3rd and 4th column are due to smaller population sizes in these groups).

When using this estimated contact matrix $\hat{\mathbf{\Delta}}_{ML}(\lambda^*)$, the estimated value of $\mathcal{R}_q(0.8)$ is 1.222, close to the estimated value in the data $\mathcal{R}_q^* = 1.216\,[95\% CI : 1.170, 1.263]$. We use the matrix $\hat{\mathbf{\Delta}}_{ML}(\lambda^*)$ as an input to the baseline simulations of the model seen in the main results.

Another way of viewing this matrix is by looking at the proportion of contacts for an individual from group j that are from group k:

$$\hat{\mathbf{Q}}(\lambda^*) := [q_{jk}] = \begin{bmatrix} 0.827 & 0.150 & 0.016 & 0.007 \\ 0.287 & 0.599 & 0.098 & 0.016 \\ 0.142 & 0.462 & 0.231 & 0.165 \\ 0.148 & 0.166 & 0.367 & 0.319 \end{bmatrix}$$

This tells us, for example, that 14.8% of the contacts of an individual from 4 come from group 1. These values are then directly used in the model to set the probability that a potential infection generated by someone from group j is from group k.

In the following section, we describe in detail the maximum likelihood process used to estimate $\hat{\mathbf{\Delta}}_{ML}(\lambda_0)$ for each of the candidate values λ_0 of the work factor. Then, in Section

S1.3.3 we describe the how we calculate the value of $\mathcal{R}_q$ both in the data (to calculate $\mathcal{R}_q^*$) and in the models (to calculate $\mathcal{R}_q(\lambda_0)$. Finally, in Section S1.3.4 we describe the "mobility matching" process, in which we allow the $\mathbf{\Delta}$ matrix to be scaled by a time-varying constant over the course of the epidemic in order to account for changes in mobility in Bogotá.

S1.3.2 Maximum likelihood process for contact matrix

The aim of the following maximum likelihood process is to estimate the symmetric contact matrix $\mathbf{\Delta}$, where the element c_{jk} describes the *total* number of out-of-home contacts (across the whole population) between individuals from group $j \in \{1, 2, 3, 4\}$ and individuals from group $k \in \{1, 2, 3, 4\}$ over the course of an average infectious period:

$$\mathbf{\Delta} := \begin{bmatrix} c_{11} & c_{12} & c_{13} & c_{14} \\ c_{21} & c_{22} & c_{23} & c_{24} \\ c_{31} & c_{32} & c_{33} & c_{34} \\ c_{41} & c_{42} & c_{43} & c_{44} \end{bmatrix}$$

Let μ_j be the mean number of out-of-home contacts for an individual from group j across all groups during an average infectious period. For the purposes of this estimation process, we assume that $\boldsymbol{\mu}(\lambda_0) := [\mu_1(\lambda_0), \mu_2(\lambda_0), \mu_3(\lambda_0), \mu_4(\lambda_0)]'$ is given, because it has been calculated in step (1a) in the previous section using a candidate value for the work factor of λ_0. What follows is thus the estimation process for $\widehat{\mathbf{\Delta}}_{ML}(\lambda_0)$. We also treat as given $\mathbf{n} := [n_1, \ldots, n_4]'$ where n_j is the number of individuals in group j in the population, because this is known from the data. Given $\boldsymbol{\mu}(\lambda_0)$ and $\mathbf{n}$, we use maximum likelihood to estimate the 6 remaining parameters of $\mathbf{\Delta}$, which we denote using the vector $\mathbf{d} = [d_{11}, d_{12}, d_{13}, d_{22}, d_{23}, d_{33}]'$.

The values c_{jk} are filled out by the parameter vectors $\boldsymbol{\mu}(\lambda_0), \mathbf{n}$, and $\mathbf{d}$. In particular, we define $\mathbf{\Delta}(\mathbf{d}, \boldsymbol{\mu}(\lambda_0), \mathbf{n})$ as the matrix with elements c_{jk} defined in the following way:

1. First, the $\mathbf{d}$ vector directly specifies some elements of the $\mathbf{\Delta}$ matrix, so that:

$$c_{jk} = d_{jk} \quad \text{for all } d_{jk} \in \mathbf{d}$$

2. Then, we enforce symmetry because contacts between each group are symmetrical so happen at the same rate, so set :

$$c_{kj} = d_{jk} \quad \text{for all } d_{jk} \in \mathbf{d}$$

3. Finally, the last row and column are pinned down by the μ vector and the population size since the sum of each row j in the $\mathbf{\Delta}$ matrix needs to sum to $\mu_j n_j$:

$$\sum_{k=1}^{4} c_{jk} = \mu_j(\lambda_0) n_j$$

$$\Rightarrow c_{j4} = \mu_j(\lambda_0) n_j - \sum_{k=1}^{3} c_{jk} \quad \text{for all } j \in \{1, 2, 3, 4\}$$

To understand how we estimate the vector $\mathbf{d}$, note first that the set of vectors $\{\mathbf{d}, \boldsymbol{\mu}(\lambda_0), \mathbf{n}\}$ also pin down the probability q_{ik} that a given contact of a person from group i is from group j, defined in the following way: $q_{jk}(\mathbf{d}, \boldsymbol{\mu}(\lambda_0), \boldsymbol{n}) := \Pr($ Secondary contact is from group k | Individual is from group $j)$

$$= \frac{[\boldsymbol{\Delta}(\mathbf{d}, \boldsymbol{\mu}(\lambda_0), \mathbf{n})]_{jk}}{\mu_j(\lambda_0) n_j}$$

We can use this probability to define the conditional likelihood function for d given the contact tracing data we observe, and the known parameters $\boldsymbol{\mu}(\lambda_0)$ and $\mathbf{n}$. The contact tracing data is a set of observations of contacts from one group to another $Z = \{(x_1, y_1), (x_2, y_2), \ldots, (x_M, y_M)\}$, where $x_m \in \{1, 2, 3, 4\}$ denotes the group of an surveyed infected individual and $y_m \in \{1, 2, 3, 4\}$ denotes the group of the reported contact (both defined for $m \in \{1, \ldots, M\}$). (Each surveyed individual can have multiple reported contacts, although this does not affect the estimation procedure.) The conditional probability of observing a given observation pair (x_m, y_m) is given by:

$$\Pr\big(Y = y_m \mid X = x_m; \mathbf{d}, \boldsymbol{\mu}(\lambda_0), \mathbf{n}\big) = q_{x_m, y_m}(\mathbf{d}, \boldsymbol{\mu}(\lambda_0), \mathbf{n})$$

So, assuming that the observations are independently and identically distributed, the conditional likelihood of observing the entire dataset is:

$$\mathcal{L}(\mathbf{d}; \boldsymbol{Z}, \boldsymbol{\mu}(\lambda_0), \mathbf{n}) = \prod_{m=1}^{M} q_{x_m, y_m}(\mathbf{d}, \boldsymbol{\mu}(\lambda_0), \mathbf{n})$$

Therefore, to run the maximum likelihood estimation procedure, we input the values of $\mu_j(\lambda_0)$ from the procedure described in Section S1.3.1, along with the known values of n. We then follow the maximisation programme below, maximising the log likelihood function while imposing the constraint that all elements of $\boldsymbol{\Delta}$ are positive:

$$\max_{\boldsymbol{k}} \quad \ln \mathcal{L}(\mathbf{d}; \boldsymbol{Z}, \boldsymbol{\mu}(\lambda_0), \mathbf{n}) \quad \text{s.t.} \quad [\boldsymbol{\Delta}(\mathbf{d}, \boldsymbol{\mu}(\lambda_0), \mathbf{n})]_{ij} > 0 \quad \forall i, j$$

We use the *nloptr* package in R [11] to run this optimization numerically, using a local COBYLA (Constrained Optimization BY Linear Approximations) algorithm. This yields a maximum likelihood estimate of the full symmetric contact matrix, which we denote $\widehat{\boldsymbol{\Delta}}_{ML}(\mathbf{d}, \boldsymbol{\mu}(\lambda_0), \mathbf{n})$, sometimes denoted $\widehat{\boldsymbol{\Delta}}_{ML}(\lambda_0)$ for brevity.

S1.3.3 Calculating $\mathcal{R}_q$

Here we calculate the value of $\mathcal{R}_q$, which we define as the average number of secondary infections generated by an infected individual at the start of the generalized quarantine period under the assumption that the proportion of susceptible individuals is 1 (or very close to 1). We treat

Figure S7. Calculation of exponential growth rate of cases in early stage

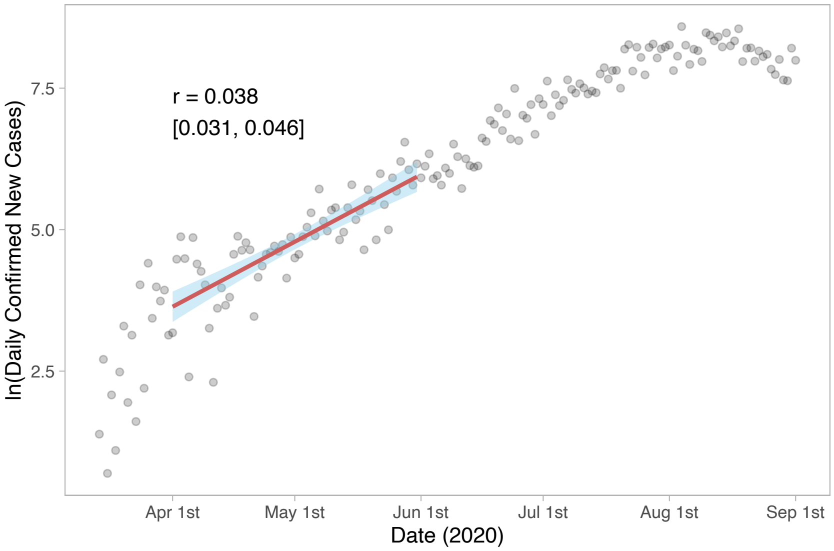

this value as a constant in the early phase of the quarantine. To calculate the value of $\mathcal{R}_q$, we use the Lotka-Euler equation [12]. This assumes exponential growth in new cases, assumes that all individuals in the population are susceptible ($S = 1$), and uses the rate of exponential growth in new cases r and the distribution of the generation interval $g(a)$ to calculate an estimate of $\mathcal{R}_q$.

First, we calculate the rate of exponential growth in new confirmed cases per day by running an OLS regression with the natural log of daily confirmed cases as the outcome variable, and the date as the independent variable. We limit our sample to the early period of the epidemic April 1st 2020 to June 1st 2020, when the exponential growth curve fits the data well and when immunity is unlikely to play a role in case growth because $S \approx 1$. This yields an estimate of r = 0.038 (95% CI: 0.031, 0.046). Figure S7 displays the log daily confirmed new cases in Bogotá over time (the gray dots), and plots the line of best fit (in red) whose slope is equal to r.

Then, we take the estimated generation interval from Figure S4, denoted $g(a)$ where a is the number of days since infection and use this to calculate the initial value of $\mathcal{R}_q$ using the Lotka-Euler equation [12]:

$$\frac{1}{\mathcal{R}_q} = \int_{a=0}^{\infty} e^{-ra} g(a) da$$

Where $g(a)$ is the density of the generation interval as a function of the day since infection a, r is the rate of exponential growth in new cases.

Using the values of $r = 0.38$ and the $g(a)$ function from Figure S4, this yields an estimate of $\mathcal{R}_q^* = 1.216\,[95\% CI : 1.170, 1.263]$. Note that this estimate comes during a period of strict lockdown in Bogotá, which explains why our estimate of $\mathcal{R}_q$ is significantly lower than the estimates of R_0 seen in the literature (which are typically calculated in conditions of full mobility

[13]).

When calculating the $\mathcal{R}_q(\lambda_0)$ values based on the model simulations, we carry out exactly the same steps apart from the fact that we add simulation fixed effects to the OLS regression for calculating r. This ensures that between-simulation variation in new cases does not affect the estimates of the rate of the exponential growth in new cases.

S1.3.4 Mobility calibration

In some model specifications, we allow for changes in the number of out of home contacts over time in the model, in order to account for changes in the level of mobility over time in Bogotá (in particular due to changes in policies such as stay-at-home orders and other mobility restrictions).

To do this, we allow for a generalized mobility factor $m(t)$ to change over time throughout the epidemic, so that at each time t, the contact matrix input into the model is equal to:

$$\hat{\mathbf{\Delta}}_{ML}(\lambda^*) \ \times m(t)$$

$m(t)$ scales the contacts of all groups similarly, and does not lead to differential mobility changes across groups through the course of the epidemic.

We estimate $m(t)$ by using an iterative process to match the model predictions to the observed pattern of new cases seen in Bogotá. This matching process calibrates the total confirmed new cases in the model (summing all groups) to the total confirmed new cases in the data (summing all groups). A case is deemed confirmed when an individual receives a positive test result (both in the model and the data). This means that any inequality between groups is a result of the other parameters we input into the model described elsewhere. The precise quantity used to match is the total (all group) per capita new confirmed cases per day, smoothed by taking a 2-week rolling average. Call this quantity it $X_k(t; m(t))$ when it is calculated from an individual simulation k. And call this same quantity $Y(d)$ when it is calculated from the data with dates d.

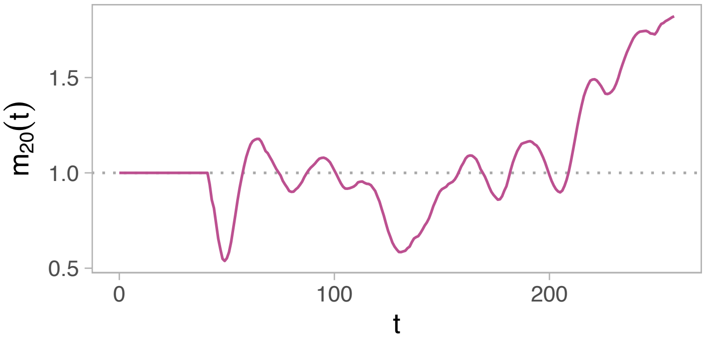

Figure S8. **Results of mobility calibration process**

The iterative matching process follows the steps below:

1. Let $m_1(t)$ be 1 for all periods t.
2. For iteration l in 1:20:
   (a) Run a set of *100* simulations with the postulated values for $m_l(t)$.
   (b) Calculate the mean across all simulations of the smoothed number of per capita new confirmed cases per day for each time t:

   $$\overline{X}_k(t; m_l(t)) := \frac{1}{50} \sum_{k=1}^{50} X_k(t; m_l(t))$$

   (c) Match the model periods $t \in \{ 0, 1, \dots \}$ to real dates d by choosing a lower bound date early in the epidemic (01/06/2020) and finding the time when new cases per day is equal in the model and the data, i.e. when Y (d = 01/06/2020) = $\overline{X}_k(t; m_l(t))$. This defines the quantities $Y(t)$.
   (d) For all t after t corresponding to the lower bound date, calculate the deviation quantity $\Delta(t)$ that indicates the extent to which the model predictions deviate from the data:

   $$\Delta(t) = \ln \left[\overline{X}_k(t; m_l(t)) \right] - \ln \left[Y(t) \right]$$

   To avoid sharp changes in mobility, we calculated a smoothed version of this function $\widetilde{\Delta}(t)$ by taking the 1 week rolling average of the deviations.
   (e) Making use of the fact that the average time from infection to receiving positive test results is approximately 3 weeks, we set the value of $m_{l+1}(t)$ in order to account for the deviations from the 3 weeks later:

   $$m_{l+1}(t) = m_l(t) - \gamma \cdot \widetilde{\Delta}(t \ + 21)$$

   where the adjustment factor γ was chosen to be 1/3, so that a 1 log deviation at $t+21$ would lead to an adjustment of 1/3 on the value of $m_{l+1}(t)$. We then use this value of $m_{l+1}(t)$ as the input to step (a) for loop $l + 1$.
3. Use $m_{20}(t)$ as the final value for $m(t)$ in the "mobility change" models.

The results of this mobility-matching process, i.e. the values of $m_{20}(t)$, are plotted in Figure S8.

S2 Figures

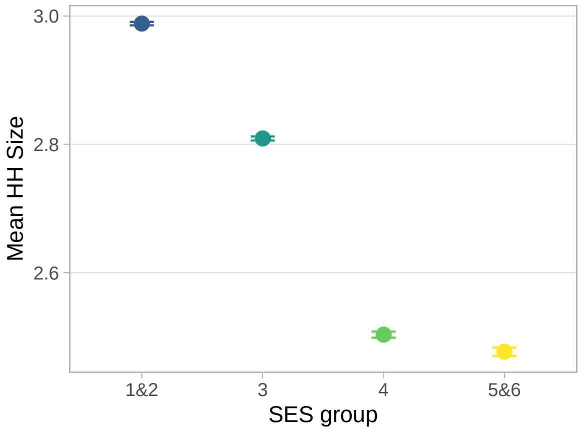

(a)

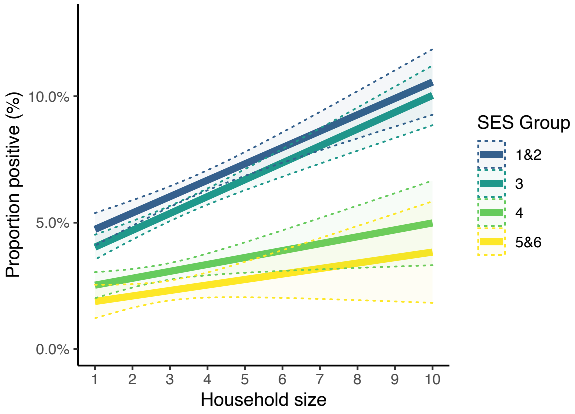

(b)

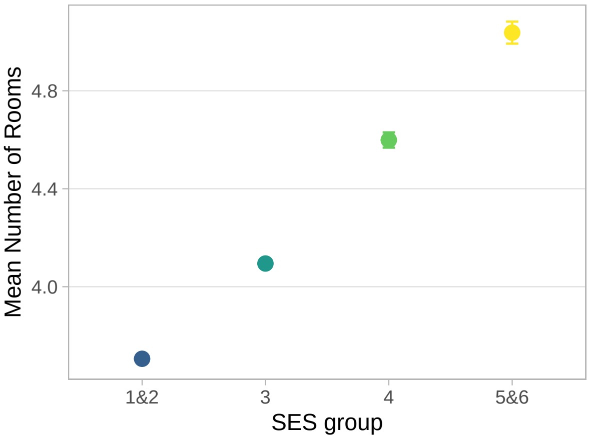

(c)

Figure S9. **Variation in within-household conditions across SES.** Panel (a) shows the mean household size for an individual in each SES. Panel (b) shows the mean number of rooms in the household of an individual in each SES. Both (a) and (b) are derived from census data. 95% confidence intervals are too small to be seen at this scale; all differences in means are significant at the 1% level. Panel (c) shows the linear fit of the relationship between household size and the probability of being infected conditional on being tested in the CoVIDA data by SES, with 95% shaded confidence intervals. The slopes of the effect of household size on positivity for strata 1&2, 3, 4 and 5&6 are 0.56, 0.58, 0.13 and 0.13 respectively, The p-value of the F-test of difference between these slopes is p=0.0033

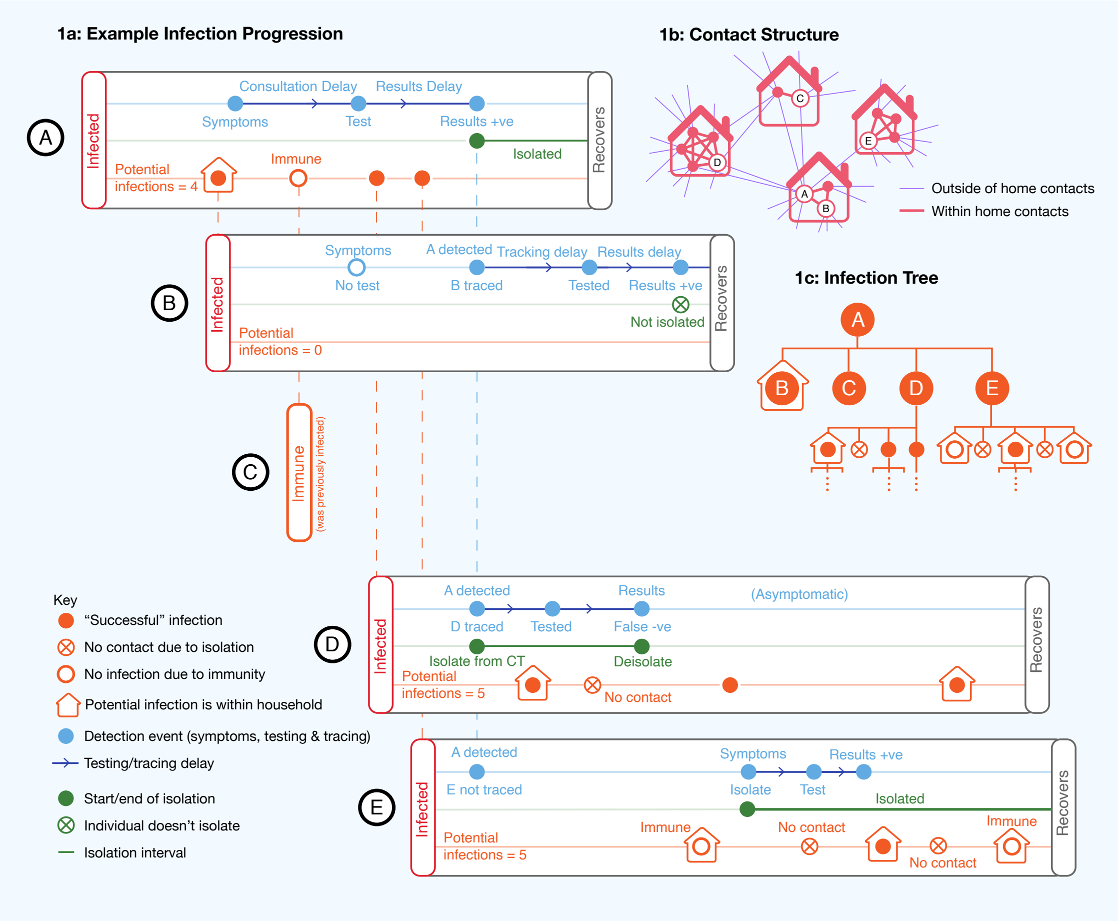

Figure S10. **Visual representation of the theoretical model**. An initial infection A potentially infects four other individuals, called B, C, D, and E. **1a:** A successfully infects B, D, and E. C does not get infected by A because she has already been infected previously, and is thus immune to further infections. A gets tested upon experiencing symptoms, and isolates upon receiving a positive test result. This begins a process of contact tracing, through which B and D (but not E) are tested. Individuals in the model may or may not be symptomatic, get tested, be contact traced, and they may isolate for a variety of reasons. **1b:** Each individual has contacts both within the home and outside the home. Every individual within the home is a contact, and the number of outside-of-home contacts is drawn from a negative binomial distribution. A contact becomes a potential infection with a probability equal to the secondary attack rate, which differs for within-home and outside-of-home contacts, and by SES. **1c:** The infection tree summarises the “branching process” in the model, i.e. the first and second generation potential infections caused by A.

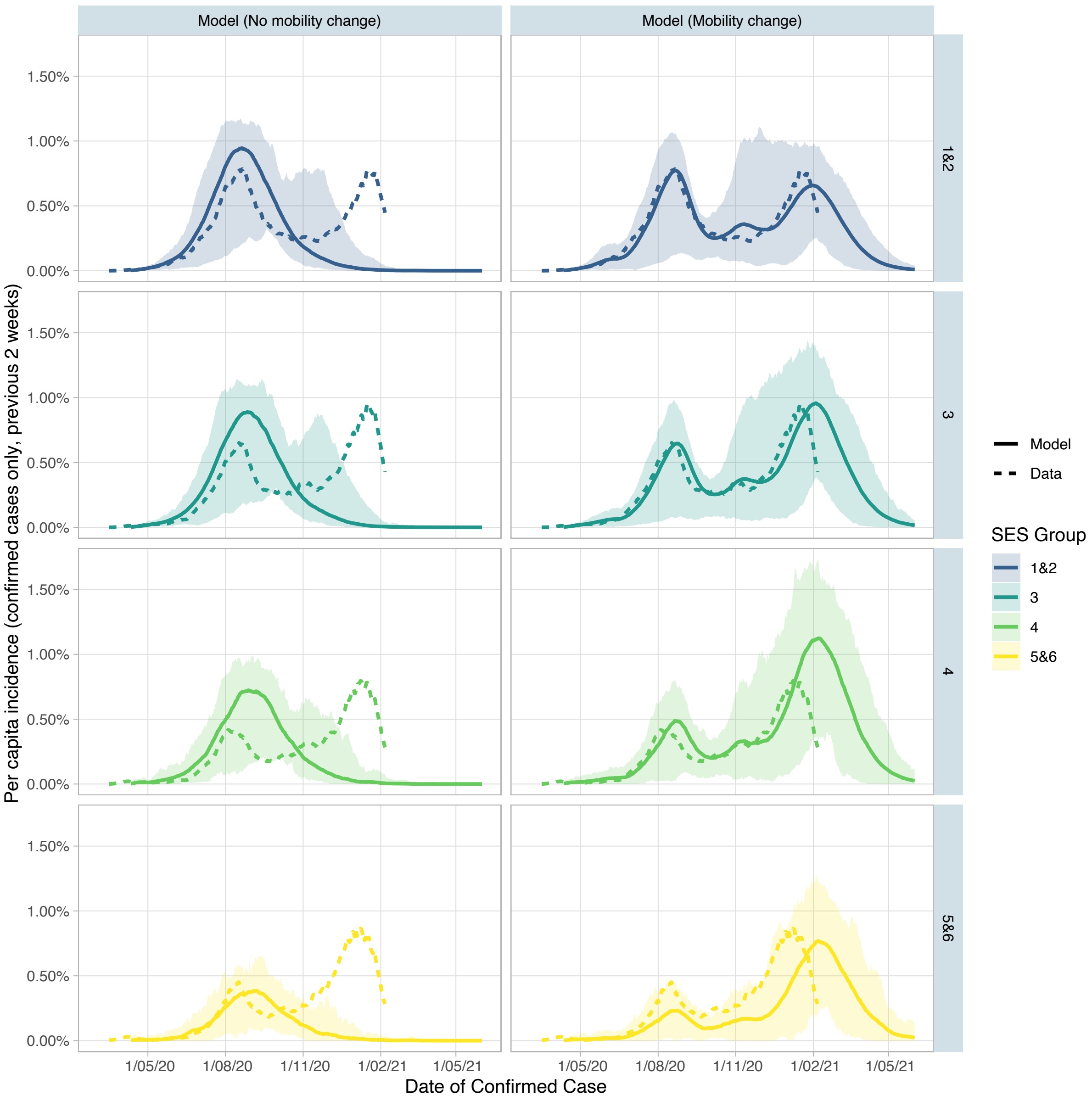

Figure S11. **Model match based on confirmed cases with confidence intervals.** This shows the same results as panels (a), (b) and (c) in Figure 2. The dashed lines represent the observed data on confirmed cases from the Health Secretary of Bogotá (HSB). The solid lines represent the median predictions over 50 model simulations of per capita confirmed incidence. The shaded intervals represent the 0.025 and 0.975 quantiles of the per capita confirmed incidence over these same 50 model simulations.

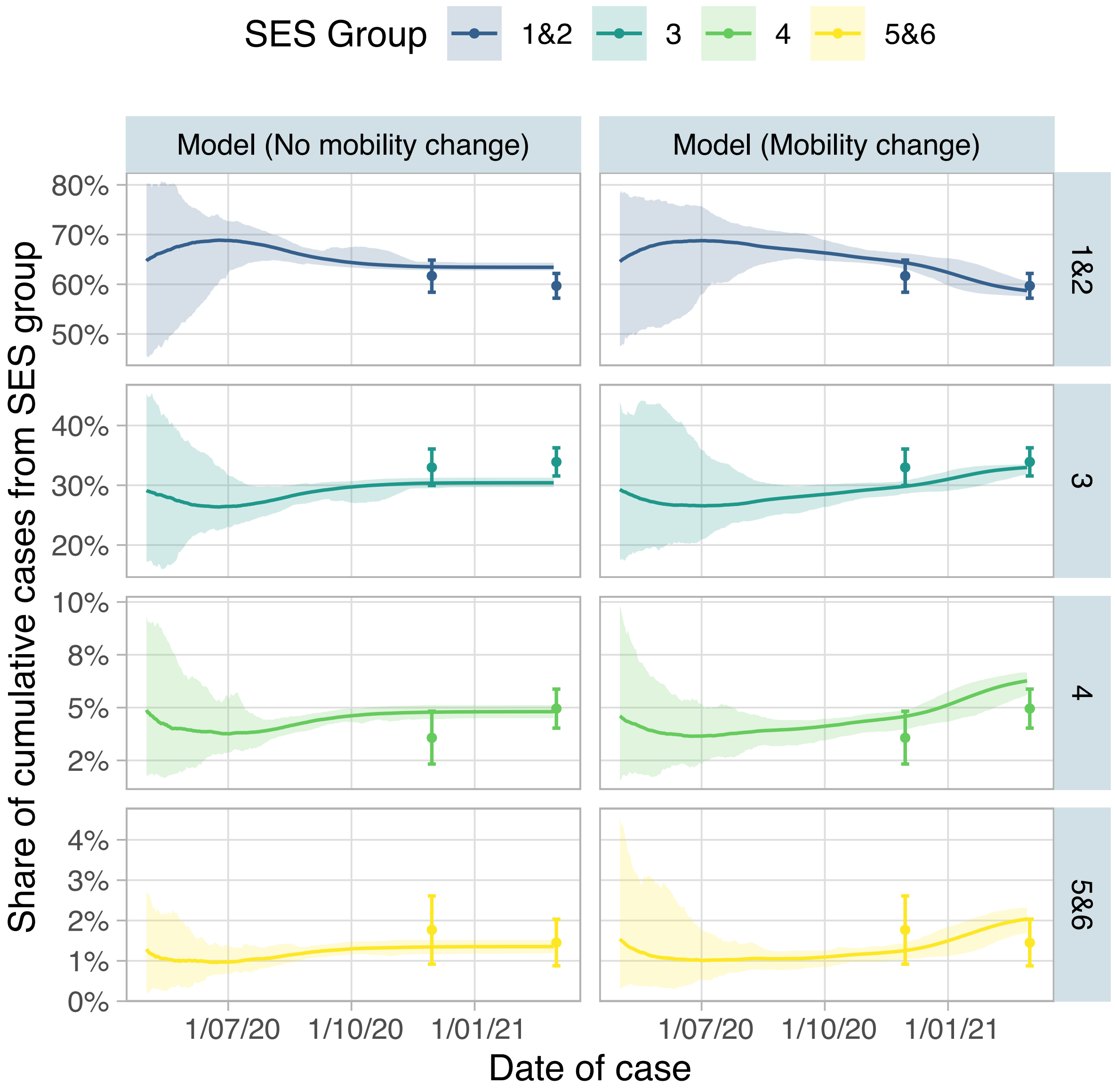

Figure S12. **Share of cases from each group**. The y-axis denotes the number of cumulative cases from the specific SES shown divided by the total number of cumulative cases across all groups. The solid line is the median, and the shaded areas denote the 0.025 and 0.975 quantiles of the model results. The points and surrounding error bars are estimations from the CoVIDA data, calculated using the methodology described in subsection S1.1.

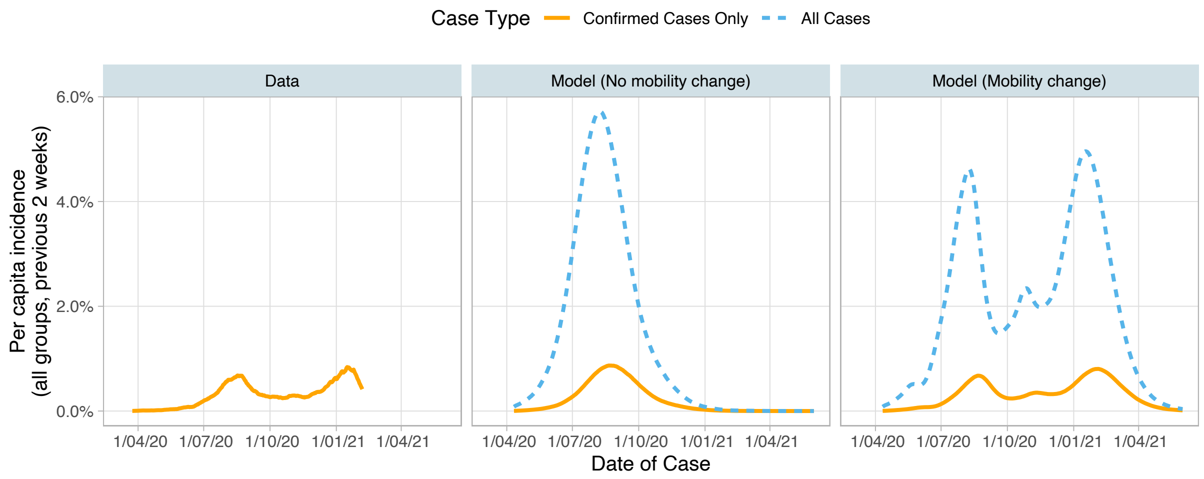

(a)

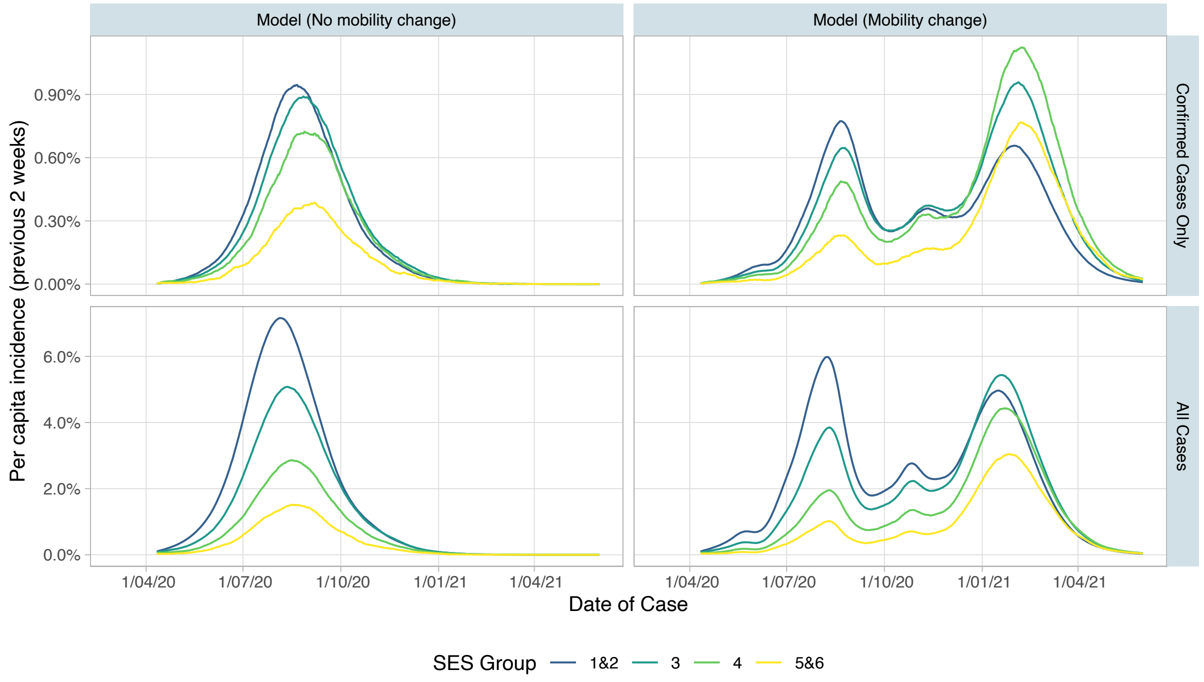

(b)

Figure S13. **Detected cases compared to confirmed cases**. Panel (a) shows the per capita incidence over the last 2 weeks for confirmed cases (orange solid line) compared to all cases (blue dashed lane). Data comes from the Health Secretary of Bogotá. Panel (b) shows the per capita incidence by SES in the model simulations, using confirmed cases in the top row and all cases in the bottom row. Note the differing scales on the Y-axes. All model results are the median for 50 simulations.

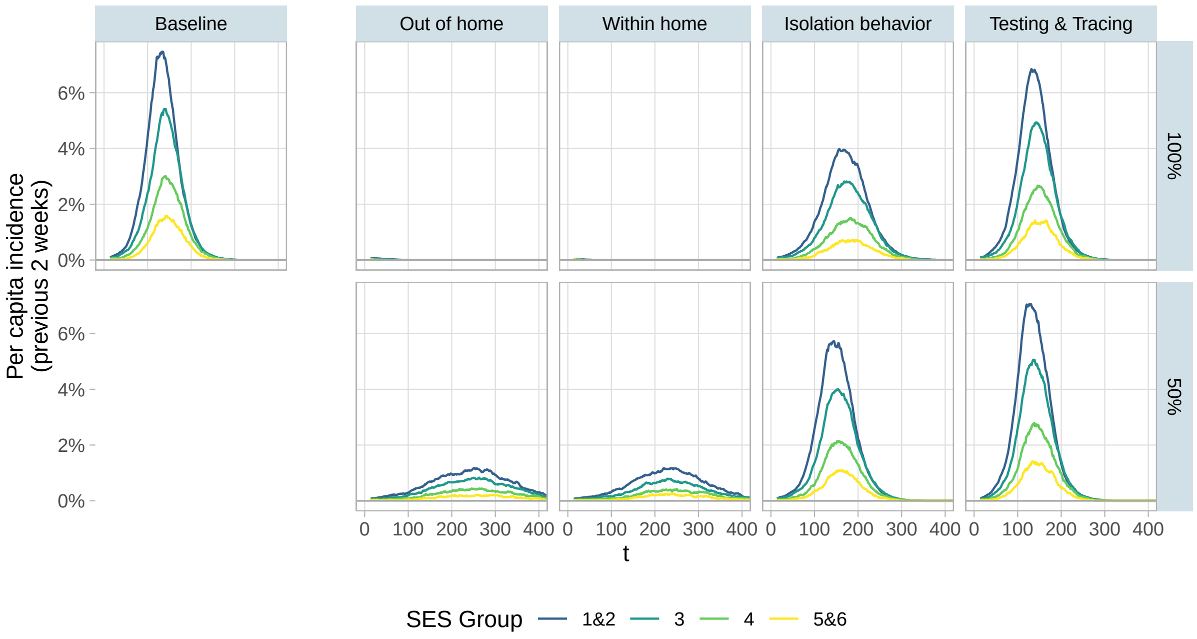

Figure S14. **Upward adjustment scenarios epidemic curves**. Additional results of the upward-adjustment scenarios, in which the parameters of all SES are adjusted to match the values of SES 5&6. The variable on the y-axis is the per capita incidence over the last 2 weeks in each SES. Parameters adjusted in each set are as follows: *out of home* (number of contacts outside the home), *within home* (within-household SAR, household size), *isolation behavior* (probability of isolating conditional on observing symptoms, testing positive, being contact traced, and probability of quarantining as a household), *testing & tracing* (probability of self testing, delay in test consultation, delay in test results, and probability of being contact traced).

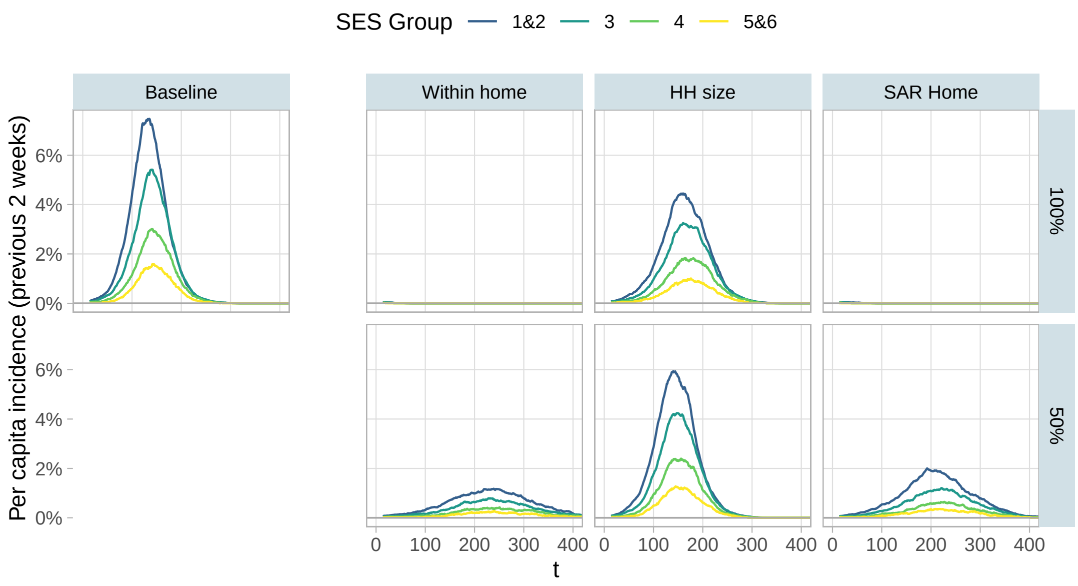

(a)

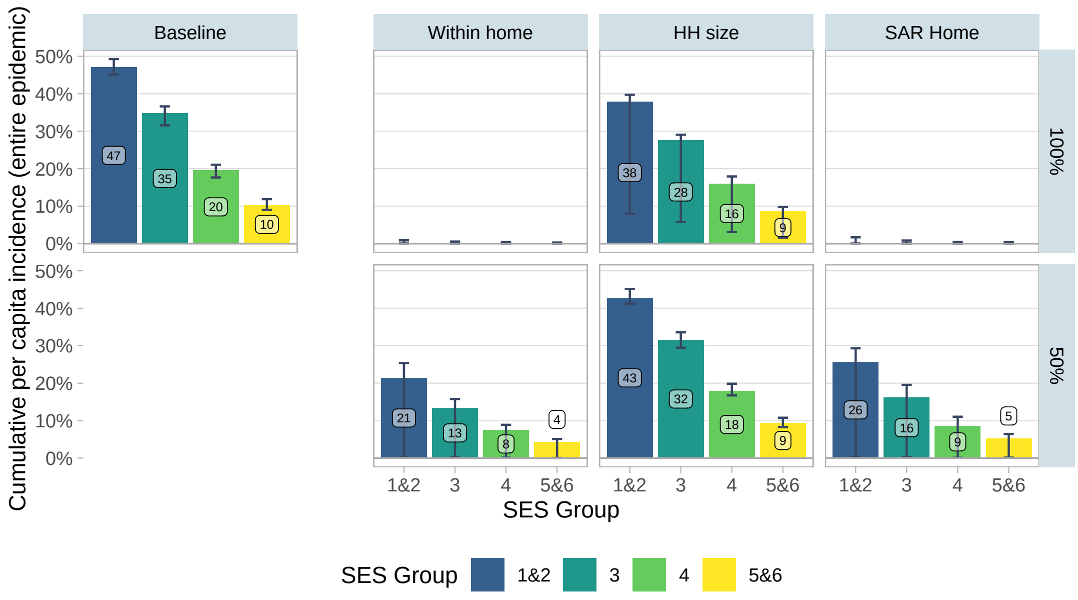

(b)

Figure S15. **Upward adjustment scenarios when changing within-home SAR and household size**. Results of additional upward-adjustment scenarios, in which the parameters of all SES are adjusted to match the values of SES 5&6. "Within-home" adjusts both household size and within-home SAR. "HH size" and "SAR Home" adjust only the single named parameter at a time. Panel (a) shows the two-weekly incidence for each group in each of the upward adjustment scenarios. Panel (b) shows the overall cumulative incidence for each group in these scenarios.

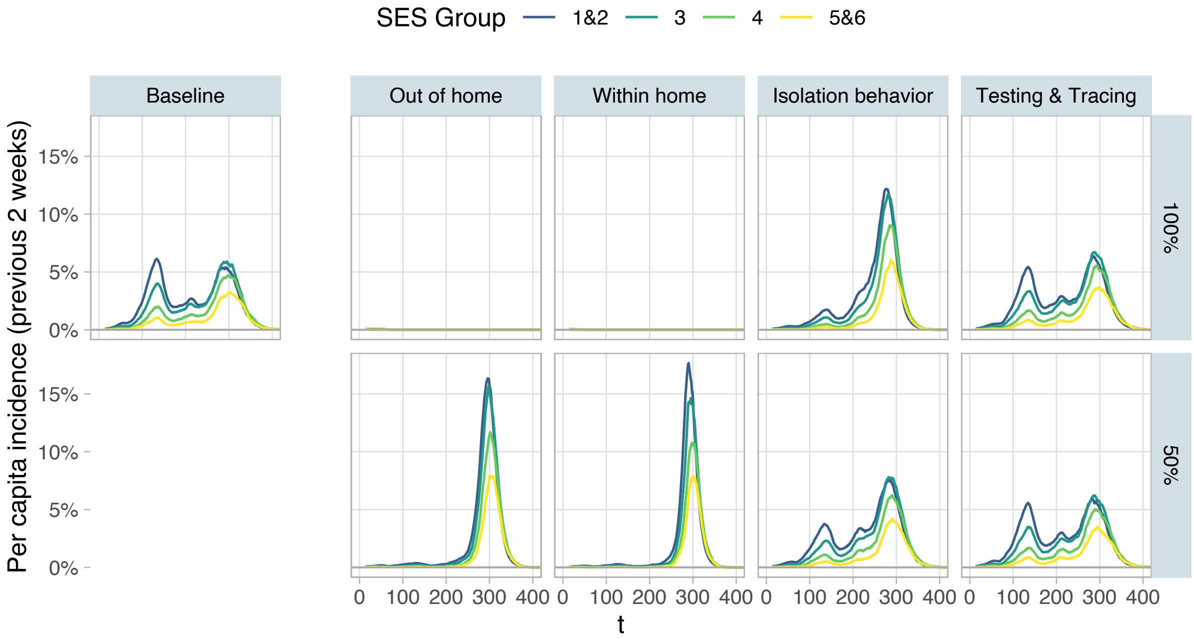

(a)

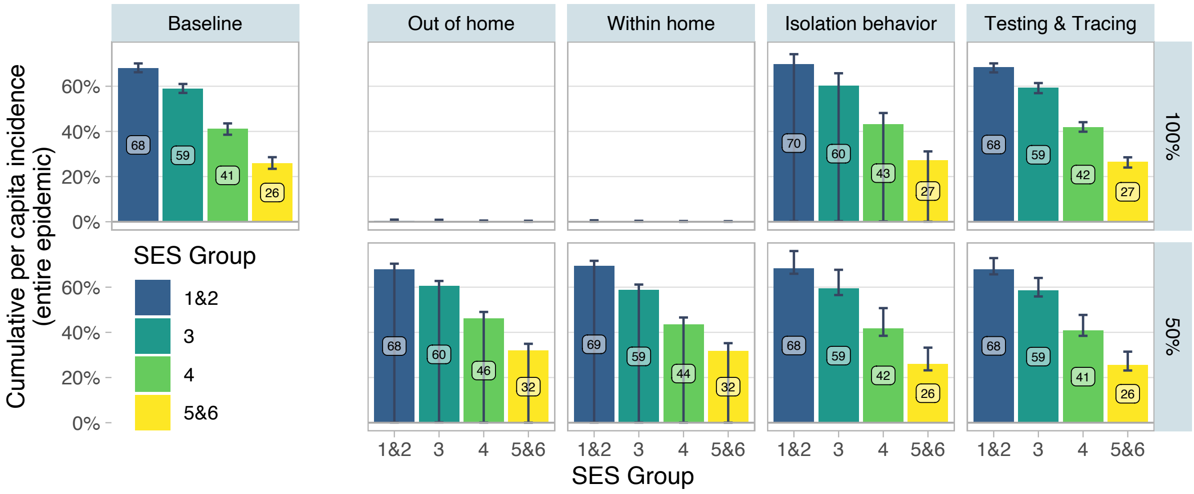

(b)

Figure S16. **Upward adjustment scenarios with mobility change**. Panel (a) shows the two-weekly incidence for each group in each of the upward adjustment scenarios. Panel (b) shows the overall cumulative incidence for each group in these scenarios. Apart from in simulations where the epidemic is completely contained (e.g. Out of home (100%) and Within home (100%)), reductions in transmission early on in the epidemic lead to much more severe second waves, leading to little overall change in the overall cumulative incidence.

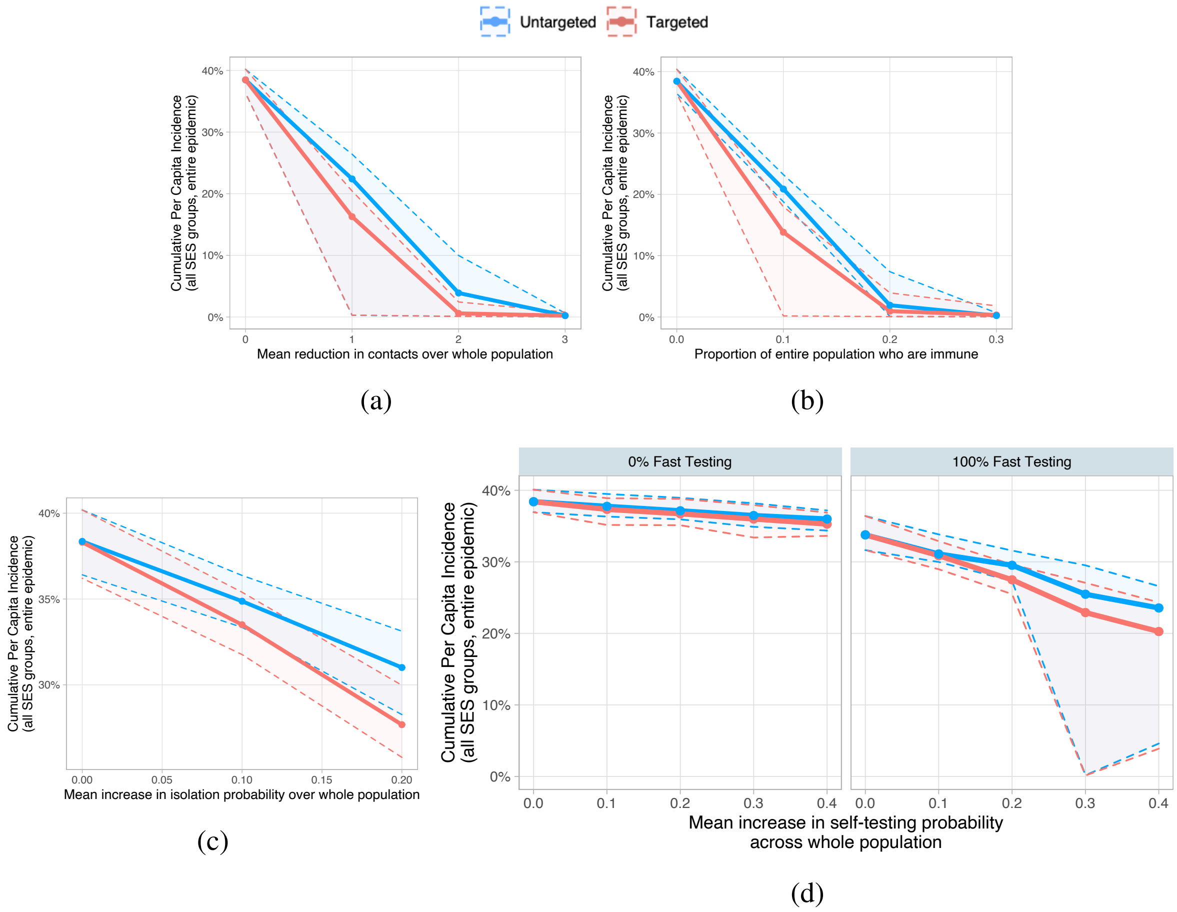

Figure S17. **Targeted and untargeted policies with varying intensity**. The outcome variable in all cases is the cumulative per capita incidence over the course of the whole epidemic for models with no mobility change. In “Targeted” scenarios, only the parameters of SES 1&2 are adjusted, but adjustments in this group are greater, such that the mean adjustment across the whole population is the same as in the untargeted scenario. Panel (a): outside-of-home contacts are reduced by 1, 2, and 3 relative to baseline scenario. Panel (b): 10%, 20% and 30% of the population are immune to the virus from the start of the epidemic. Panel (c): mean increase of 10 and 20 percentage points in select isolation parameters (probability of isolating conditional on being symptomatic and being contact traced). Panel (d): mean increase of 10, 20, 30, and 40 percentage points in the probability of being tested after observing symptoms. 0% fast testing indicates the same testing delays as in the baseline scenario. 100% fast testing indicates that all tests have a consultation delay of 1 day and a results delay of 1 day. The estimates represent the median of 50 simulations, while the confidence intervals represent the 0.025 and 0.975 quantiles of these simulations.

S3 Tables

Table S1. Self-declared prevention practices

Dependent variable:	No Trips (last 14 days)	Frequency of hand washing	Duration of hand washing	Frequency of mask usage	Frequency of antibacterial usage
Stratum 3	-0.041***	0.028***	0.0093	-0.034***	-0.00078
	[0.0029]	[0.0093]	[0.0088]	[0.0050]	[0.0085]
Stratum 4	-0.093***	0.060***	0.0065	-0.095***	-0.066***
	[0.0040]	[0.011]	[0.011]	[0.0068]	[0.010]
Strata 5&6	-0.13***	0.051***	-0.045***	-0.13***	-0.067***
	[0.0054]	[0.014]	[0.013]	[0.0090]	[0.013]
Constant	0.89***	2.83***	3.00***	3.77***	2.78***
(Mean SES 1&2)	[0.0021]	[0.0072]	[0.0069]	[0.0037]	[0.0066]
Observations	74,345	74,613	74,612	74,544	74,467
p-value of F-test of joint significance	0	0.0000005	0.00016	0	0

Robust standard errors in brackets. *** p<0.01, ** p<0.05, * p<0.1, all p-values and F-test are double-sided tests. Linear regressions. The outcome variable “No trips” is a dummy indicating that the individual didn’t take any trips in the 14 days preceding the survey. Outcome variables in columns 2 to 5 are transformed into an index from 1 to 4 where higher values of the outcome variable always means higher level of protection. The last line presents the p-value of the joint significance of the three SES dummies in the regression, hence it tests the nul hypothesis that the average value of the outcome variable is the same for all SES.

Table S2. Number of days worked outside home as a function of sickness and SES

	Days worked outside home (in last 14 days)
Stratum 3	-0.65*
	[0.33]
Stratum 4	-1.12***
	[0.39]
Strata 5&6	-2.13***
	[0.46]
Strata 1&2 × fraction sick	-1.92***
	[0.52]
Stratum 3 × fraction sick	-2.01***
	[0.37]
Stratum 4 × fraction sick	-1.43**
	[0.57]
Stratum 5&6 × fraction sick	-1.32
	[0.83]
Constant	4.95***
(Mean for SES 1&2 if 0 days sick)	[0.27]
Observations	6,798

Robust standard errors in brackets. *** p<0.01, ** p<0.05, * p<0.1, all p-values and F-test are double-sided tests. The sample is restricted to individuals that have been sick for at least one day and observations are weighted by occupation. Linear regression where the explained variable is the respondent's answer to the question "In the last 14 days, how many days have you worked outside of home?". "fraction sick" is equal to the number of days with symptoms in the past 14 days / 14; it goes from 0 to someone who had no symptoms to 1 for someone who had symptoms during the past 14 days.

Table S3. Statistical predictions of the number of days worked using results from Table S2

Stratum	Prediction if 0 days sick	Prediction with 14/14 days sick
1 & 2	4.95	3.03
3	4.30	2.29
4	3.83	2.40
5 & 6	2.82	1.50

Example of calculations of statistical linear predictions, for stratum 3: 4.95 -0.65 = 4.30 provides the average number of days worked for a person in stratum 3 if the person was sick during 0 days out of 14. 4.95 -0.65 -2.01 =4.30 provides the average number of days worked for a person in stratum 3 if the person was sick during 14 days out of 14. The difference (-2.01) is the effect of being 14 days sick on the number of days worked for stratum 3.

Table S4. Model parameters common across all SES

**Model Parameter**	**Value**	**Source**
Proportion of asymptomatic infections	20%	Buitrago-Garcia et al. (2020)
Asymptomatic Relative Risk	35%	Buitrago-Garcia et al. (2020)
Out-of-home contacts distribution	Negative binomial with k = 0.58	Bi et al. (2020)
Incubation period distribution	Lognormal with μ = 1.63 and σ = 0.5	McAloon et al. (2020)
Serial interval distribution	Gamma with α = 8.12, β = 0.64, and translated by Δx = -7.5	He et al. (2020)
Generation interval distribution	Empirical distribution matching gamma with mean of 5.2 days, shape = 4.72	Inferred from incubation and serial interval distribution
Test sensitivity distribution	Empirical distribution	Grassly et al. (2020)

Table S5. Model parameters for each SES

(a) Parameter Summary

**Model Parameter**	**SES** 1&2	3	4	5&6	**Source / Construction**
P(Self Test|Symptoms)	0.188	0.244	0.356	0.341	$d/((1-A)(1-F))$ where d = share detected among positive from Table S7, A = share of asymptomatics in the model (20%), F is the estimated share of false negatives in a typical model (22%)
P(Isolation|Symptoms)	0.526	0.520	0.261	0.395	$1-(w_s/w)$, where w_s = the avg. number of days worked after having been sick for 14 days, inferred from Table S3, and w is the avg. number of days worked
P(Isolation|Contact traced)	0.300	0.290	0.000	0.106	$1-(w_{ct}/w)$, where w_{ct} = the avg. number of days worked when individual knows about contact with an infected person We assume this is weakly greater than 0
P(Isolation|Test)	0.858	0.858	0.858	0.858	$1-(w_t/w)$, where w_t = the avg. number of days worked when an individual has received a positive test
P(Household quarantine)	0.561	0.503	0.270	0.254	$1-(w_{hh}/w)$, where w_{hh} = the avg. number of days worked when someone in the same household is tested positive (does not vary by SES)
P(Contact traced)	0.189	0.227	0.291	0.325	$c/(n+\lambda^{*}w)$, where c = average number of contacts traced from Table S7, n = Number of non-work contacts outside of home from Table 1, λ^{*} = 0.8 outside work factor as described in Section 3, and w is days worked outside of home in Table 1
SAR (Home) for symptomatics	0.303	0.310	0.270	0.121	$SAR_{hh}/(\rho A+(1-A))$ where SAR_{hh} = estimated SAR (Home) from Table S7, ρ = the relative risk of asymptomatics (0.35), A is the share of asymptomatics (20%)
SAR (Outside home) for symptomatics	0.146	0.146	0.146	0.146	$SAR_{out}/(\rho A+(1-A))$ where SAR_{out} = estimated SAR (out) from Table S7, ρ = the relative risk of asymptomatics (0.35), A is the share of asymptomatics (20%)
K matrix	[See Panel (b)]				Estimated using process seen in Section S1.3.1
Household size	[full distribution used]				Observed distribution from census data
Test consultation delay	[full distribution used]				Observed distribution from HSB data
Test results delay	[full distribution used]				Observed distribution from HSB data
Contact tracing delay	[full distribution used]				Assumed to be same for all SES groups and drawn from the observed distribution for test consultation delays

(b) K matrix

**Index Case SES**	**Contact Case SES** 1&2	3	4	5&6	Total
1&2	272,155	49,440	5,162	2,432	329,190
3	49,440	102,967	16,864	2,729	172,000
4	5,162	16,864	8,431	6,022	36,480
5&6	2,432	2,729	6,022	5,247	16,430
Total	329,190	172,000	36,480	16,430	554,100

Table S6. Age by SES group and relationship with positivity

Panel A: *Proportion of population in each age-group for each SES (census data)*

	SES Group			
Age	1&2	3	4	5&6
0-14	21.9%	16.2%	12.8%	12.4%
15-29	29.1%	25.6%	22.1%	19.6%
30-49	28.6%	30.0%	32.3%	29.8%
50-69	16.5%	21.4%	24.2%	27.0%
70+	3.9%	6.8%	8.5%	11.2%
Total:	100%	100%	100%	100%
Population size	3,673,782	2,411,701	672,543	360,532

Panel B: *OLS correlation between age and positivity rate (CoVIDA data)*

Dep var: Tested positive for COVID-19 (=1)	
Age (years)	-0.00024***
	[0.000070]
Observations	58,831

Notes: Panel A displays the proportion of the population in each age group for each stratum, source: Censo Nacional de Población y Vivienda 2018 [14]. Panel B shows the result of an OLS regression with the positivity of the PCR test in the CoVIDA data as the dependent variable, and age as the independent variable. Robust standard errors in brackets. *** p<0.01, ** p<0.05, * p<0.1.

Table S7. Potential Determinants of Infection Estimated by SES

Category of Parameters	Parameter	SES Group 1 & 2	SES Group 3	SES Group 4	SES Group 5&6	Population average	p-val of F-test of joint significance	Data Source	Estimation Method
Infections outside the home	Days working outside of home (in previous 10 working days)	6.4<br>[0.051]<br>{6.294;6.493}	4.8<br>[0.041]<br>{4.686;4.847}	3.2<br>[0.051]<br>{3.145;3.346}	2.5<br>[0.062]<br>{2.360;2.601}	4.6<br>[0.026]<br>{4.600;4.686}	<0.001	CoVIDA	Average of answers to the question "in the last 14 days, how many days have you worked outside of home", restricting answer to respondents without symptoms nor known contact with an infected person.
	Number of non-work contacts outside of home (time frame)	1.108<br>[0.096]<br>{0.919;1.296}	1.392<br>[0.080]<br>{1.235;1.548}	1.506<br>[0.152]<br>{1.207;1.805}	1.423<br>[0.281]<br>{0.860;1.986}	1.314<br>[0.056]<br>{1.222;1.406}	0.063	CoVIDA	Positive cases are traced, and their listed contacts are categorized. We count the number of contacts that are not from the same household, nor from work and take the average of that number.
	Secondary attack rate (outside of home)	15%<br>[0.022]<br>{0.108;0.193}	13%<br>[0.015]<br>{0.098;0.155}	8%<br>[0.025]<br>{0.032;0.129}	12%<br>[0.056]<br>{0.004;0.232}	0.127<br>[0.011]<br>{0.109;0.145}	0.2	CoVIDA	All listed contacts are invited for testing. We use the positivity rate of contacts from outside the household that we traced.
	Contact matrix structure			[see Panel B]				CoVIDA	To create the matrix, we use the self-declared stratum of the index cases that were traced and the stratum of their non-household contacts listed
Infections inside home	Household size	2.99<br>0.001<br>{2.99; 2.99}	2.81<br>0.002<br>{2.81; 2.81}	2.50<br>0.002<br>{2.50; 2.51}	2.48<br>0.003<br>{2.47; 2.48}	2.84<br>0.001<br>{2.84; 2.85}	<0.001	Census	Individual level average of household size (the variable is available in the census))
	Secondary attack rate (inside home)	0.263<br>[0.025]<br>{0.215;0.312}	0.27<br>[0.019]<br>{0.232;0.307}	0.235<br>[0.042]<br>{0.152;0.319}	0.105<br>[0.050]<br>{0.003;0.207}	0.258<br>[0.014]<br>{0.235;0.281}	0.02	CoVIDA	All listed contacts are invited for testing. We use the positivity rate of contacts from inside the household that we traced.
Isolation behaviour	Isolation after positive test result (% self-declared)	0.87<br>[0.01]<br>{0.85;0.89}	0.85<br>[0.01]<br>{0.84;0.87}	0.86<br>[0.02]<br>{0.83;0.89}	0.87<br>[0.02]<br>{0.82;0.92}	0.86<br>[0.01]<br>{0.85;0.87}	0.61	CoVIDA	Positive cases of the CoVIDA project are followed up and we ask them whether they were able to isolate (yes/no). We take the share of respondents who answered that they isolate.
	# of days worked when has symptoms	3.0	2.3	2.4	1.5	2.6	<0.001	CoVIDA	Answers to "In the last 14 days, how many days have you worked outside of home?", restricting answer to respondents with symptoms, and predicting the number of days of work for a person that has been infected during 14 days. (see table S3 for detailed explanation)
	# days worked when knowing about positive contact	4.5	3.4	3.5	2.2	3.9	0.016	CoVIDA	Answers to "In the last 14 days, how many days have you worked outside of home?", It assumes same ability to isolate as variable above, but rescaled to respondents with a (self-declared) contact that was tested positive.
	# days worked when someone is tested positive in same household	2.8<br>[0.137]<br>{2.535;3.072}	2.4<br>[0.104]<br>{2.167;2.573}	2.4<br>[0.172]<br>{2.031;2.706}	1.9<br>[0.199]<br>{1.458;2.242}	2.5<br>[0.071]<br>{2.349;2.582}	0.004	CoVIDA	Average answers to "In the last 14 days, how many days have you worked outside of home?", restricting answer to respondents with a (self-declared) positive person in the same household.
Testing & tracing	Share detected among positive	11.7%	15.2%	22.2%	21.3%	16.1%	-	CoVIDA and HSB	We estimated total number of cases from June 2020 to January 2021 with CoVIDA data (to predict cases in the entire population) and compare it to detected cases in the HSB data.
	Test consultation delay in days	5.56<br>(0.01)<br>{5.54; 5.58}	5.59<br>(0.01)<br>{5.57; 5.62}	5.41<br>(0.03)<br>{5.36; 5.47}	5.26<br>(0.04)<br>{5.18; 5.34}	5.55<br>(0.01)<br>{5.53; 5.57}	<0.001	HSB	Average of the difference between testing date and onset of symptoms
	Test results delay in days	3.94<br>(0.01)<br>{3.92; 3.96}	3.57<br>(0.01)<br>{3.55; 3.59}	3.28<br>(0.02)<br>{3.25; 3.32}	3.05<br>(0.03)<br>{3.00; 3.10}	3.72<br>(0.01)<br>{3.71; 3.73}	<0.001	HSB	Average of the difference between test results and testing date
	Average number of contacts traced	1.73	1.74	1.75	1.75		-	online	Accessible online, separately for affiliated and non-affiliated. We weight the values by the share of affiliated within each stratum.
	Proportion of infections that are contact traced	81%	84%	88%	89%	83%	-	online	Accessible online, separately for affiliated and non-affiliated. We weight the values by the share of affiliated within each stratum.
	Population size in Bogota	4,063,470	2,857,861	757,923	365,459	8,044,713		Census	Counting of observations per stratum
	Sample size in CoVIDA Survey Data	22,171	31,636	14,608	7,539	75,954		CoVIDA	Counting of observations per stratum
	Sample size with PCR test in CoVIDA Data	15,818	24,450	11,759	6,158	58,185		CoVIDA	Counting of observations per stratum

The table displays the same results as Table 1 and extends the information provided with standard errors [in brackets], confidence intervals in curly brackets, the data source and a an explanation of the estimation method. CoVIDA refers to the primary data collected within the CoVIDA project led by the authors, and HSB refers to administrative data of the Health Secretary of Bogotá on detected cases.

References

[1] Miller, T. E. *et al.* Clinical sensitivity and interpretation of PCR and serological COVID-19 diagnostics for patients presenting to the hospital. *The FASEB Journal* **34**, 13877–13884 (2020).

[2] Laajaj, R. *et al.* COVID-19 spread, detection, and dynamics in Bogota, Colombia. *Nature Communications* **12**, 1–8 (2021).

[3] Bi, Q. *et al.* Epidemiology and transmission of COVID-19 in 391 cases and 1286 of their close contacts in Shenzhen, China: A retrospective cohort study. *The Lancet Infectious Diseases* **20**, 911–919 (2020).

[4] Buitrago-Garcia, D. *et al.* Occurrence and transmission potential of asymptomatic and presymptomatic SARS-CoV-2 infections: A living systematic review and meta-analysis. *PLOS Medicine* **17**, e1003346 (2020).

[5] McAloon, C. *et al.* Incubation period of COVID-19: A rapid systematic review and meta-analysis of observational research. *BMJ Open* **10**, e039652 (2020).

[6] He, X. *et al.* Temporal dynamics in viral shedding and transmissibility of COVID-19. *Nature Medicine* **26**, 672–675 (2020).

[7] Azzalini, A. & work(s):, A. D. V. R. The Multivariate Skew-Normal Distribution. *Biometrika* **83**, 715–726 (1996).

[8] Lee, S. X. & McLachlan, G. J. EMMIXuskew: An R Package for Fitting Mixtures of Multivariate Skew t Distributions via the EM Algorithm. *Journal of Statistical Software* **55** (2013).

[9] Challen, R., Brooks-Pollock, E., Tsaneva-Atanasova, K. & Danon, L. Meta-analysis of the SARS-CoV-2 serial interval and the impact of parameter uncertainty on the COVID-19 reproduction number. *medRxiv* (2020). URL `https://www.medrxiv.org/content/early/2020/11/20/2020.11.17.20231548`. `https://www.medrxiv.org/content/early/2020/11/20/2020.11.17.20231548.full.pdf`.

[10] Grassly, N. C. *et al.* Comparison of molecular testing strategies for COVID-19 control: A mathematical modelling study. *The Lancet Infectious Diseases* S1473309920306307 (2020).

[11] Johnson, S. G. The nlopt nonlinear-optimization package, v1.2.2.2 (2020).

[12] Wallinga, J. & Lipsitch, M. How generation intervals shape the relationship between growth rates and reproductive numbers. *Proceedings of the Royal Society B: Biological Sciences* **274**, 599–604 (2007).

[13] Hilton, J. & Keeling, M. J. Estimation of country-level basic reproductive ratios for novel Coronavirus (SARS-CoV-2/COVID-19) using synthetic contact matrices. *PLoS computational biology* **16**, e1008031 (2020).

[14] DANE, D. National population census and housing 2018 (2018). URL `https://www.dane.gov.co/index.php/en/estadisticas-por-tema/demografia-y-poblacion/censo-nacional-de-poblacion-y-vivenda-2018`.
